# A general approach for predicting protein epitopes targeted by antibody repertoires using whole proteomes

**DOI:** 10.1371/journal.pone.0217668

**Published:** 2019-09-06

**Authors:** Michael L. Paull, Tim Johnston, Kelly N. Ibsen, Joel D. Bozekowski, Patrick S. Daugherty

**Affiliations:** Department of Chemical Engineering, University of California Santa Barbara, California, United States of America; Albert Einstein College of Medicine, UNITED STATES

## Abstract

Antibodies are essential to functional immunity, yet the epitopes targeted by antibody repertoires remain largely uncharacterized. To aid in characterization, we developed a generalizable strategy to predict antibody-binding epitopes within individual proteins and entire proteomes. Specifically, we selected antibody-binding peptides for 273 distinct sera out of a random library and identified the peptides using next-generation sequencing. To predict antibody-binding epitopes and the antigens from which these epitopes were derived, we tiled the sequences of candidate antigens into short overlapping subsequences of length k (k-mers). We used the enrichment over background of these k-mers in the antibody-binding peptide dataset to predict antibody-binding epitopes. As a positive control, we used this approach, termed K-mer Tiling of Protein Epitopes (K-TOPE), to predict epitopes targeted by monoclonal and polyclonal antibodies of well-characterized specificity, accurately recovering their known epitopes. K-TOPE characterized a commonly targeted antigen from *Rhinovirus A*, predicting four epitopes recognized by antibodies present in 87% of sera (n = 250). An analysis of 2,908 proteins from 400 viral taxa that infect humans predicted seven enterovirus epitopes and five Epstein-Barr virus epitopes recognized by >30% of specimens. Analysis of *Staphylococcus* and *Streptococcus* proteomes similarly predicted 22 epitopes recognized by >30% of specimens. Twelve of these common viral and bacterial epitopes agreed with previously mapped epitopes with p-values < 0.05. Additionally, we predicted 30 HSV2-specific epitopes that were 100% specific against HSV1 in novel and previously reported antigens. Experimentally validating these candidate epitopes could help identify diagnostic biomarkers, vaccine components, and therapeutic targets. The K-TOPE approach thus provides a powerful new tool to elucidate the organisms, antigens, and epitopes targeted by human antibody repertoires.

## Introduction

Immunological memory allows for rapid antibody responses towards diverse antigens long after initial exposure. For example, the adaptive immune response to many vaccinations is often sustained throughout an individual’s lifetime [1]. This immunological information is archived within the genes encoding B-cell and T-cell receptors along with the corresponding receptor structures, but has proven difficult to characterize in a comprehensive manner. The ability to more fully interrogate immunological memory could reveal exposures to pathogens, commensal organisms, and allergens. Such information has proven useful for correlating antibody responses with disease outcomes to design more effective vaccines [2]. A detailed record of immune exposures can also facilitate the identification of biomarkers to diagnose infectious [3], autoimmune [4], and allergic conditions [5]. Furthermore, the capability to broadly characterize antibody repertoires at the epitope level could be used to identify conserved pathogen epitopes [6] and tumor specific antigen epitopes [7] to aid in therapeutic discovery.

A disease with prominent antibody responses is the common viral infection HSV, which causes human infections in the orofacial region (“cold sores”) and the genital region (“genital ulcers”) [8]. In 2012, the global prevalence of HSV1 was 3.7 billion people ages 0–49 [9] and the global prevalence of HSV2 was 417 million people ages 15–49 [10]. Diagnostic discovery generally focuses on diagnosing HSV2, since HSV2 infections can exacerbate HIV infections [10]. However, HSV1 and HSV2 contain the same genes [11] and the protein-coding regions of the HSV1 and HSV2 genomes share 83% sequence homology [12]. Therefore, researchers have often analyzed HSV glycoprotein G, since it differs substantially between the two HSV species [13]. In general, efforts have been limited to analyses of the surface-exposed envelope glycoproteins [14–17], using approaches such as microarrays [18]. Therefore, it would be novel to probe immunological memory using the entire proteomes of HSV1 and HSV2.

Immunological memory has been investigated extensively through sequencing the variable regions of B- and T-cell receptor encoding genes amplified from circulating cells [19]. These methods have proven useful for identifying receptor-encoding genes that associate with vaccination [20]. Nevertheless, such genetic information has not generally provided insight into the specific environmental antigens and epitopes targeted, unless they are known *a priori*. Furthermore, these methods require large specimen volumes (>10 mL) to obtain a sufficient quantity of cells [20]. Thus, there remains a need for methods that identify the diverse antigen targets of adaptive immunity.

Several methods have been developed to profile the protein epitopes of the secreted antibody repertoire [21]. Approaches have often focused on linear epitopes since 85% of epitopes contain at least one contiguous stretch of five amino acids [22]. By analyzing linear epitopes, researchers have identified sensitive and specific diagnostic epitopes for numerous diseases [21]. One common approach to epitope mapping is to generate short overlapping peptides by tiling candidate antigens. These peptides are then assayed for serum antibody reactivity in peptide microarray [23] or bacteriophage display library [24] formats. However, because these methods are biased towards specific organisms, they do not enable comprehensive or hypothesis-free immune evaluation. One strategy to overcome the limitations of tiling experiments is to use fully random peptide libraries [5,25,26]. Here, experiments are less biased and methods can analyze epitopes corresponding to a variety of organisms and antigens. A disadvantage of microarrays is that they are typically several orders of magnitude less diverse than peptide display libraries (e.g. 10^5^ [25] versus 10^10^ [5]), limiting the effectiveness with which current methods can achieve epitope discovery for low titer antibodies. In random library experiments, epitopes are typically discovered using *de novo* motif discovery by unsupervised clustering [27]. The most widely used algorithm for this purpose, MEME, scales approximately quadratically with the number of input sequences, making it less useful for analyzing large datasets resulting from next generation sequencing (NGS). While full-length antibody-binding peptides can be analyzed, the majority of the binding energy is typically derived from just 5–6 amino acids [28], thus other amino acids within the peptide will contribute noise. To rectify this problem researchers developed the IMUNE algorithm to reduce peptide datasets into statistically enriched patterns and cluster these patterns to build motifs [29].

A significant challenge for epitope mapping approaches is the association of epitopes and motifs with their corresponding antigens. Neither MEME nor IMUNE have the integrated capability to connect motifs to plausible antigens. Also, motifs identified through these methods often fail to reach the seven amino acids requirement for unambiguous identification of antigens within the full database of protein sequences [30]. Fundamentally, linear stretches in epitopes are typically less than seven amino acids in length [22], therefore, protein database searches of individual epitopes (such as through BLAST [31]) often fail to achieve statistical significance. Using multiple epitope matches within a single candidate antigen can increase the confidence of antigen prediction [26,32]. However, this method is insufficient for antigens with a single important epitope. Additionally, protein database searches are conducted using short amino acid sequences, therefore these searches do not fully leverage large quantitative binding datasets. To address these challenges, we present a general approach for associating epitopes with antigens using large peptide datasets. The K-mer Tiling of Protein Epitopes (K-TOPE) algorithm identifies epitopes by computationally tiling candidate antigens into k-mers, which are then evaluated within large datasets of antibody-binding peptides. Here, we demonstrate the utility of this approach by predicting linear epitopes within the proteomes of several prevalent infectious pathogens.

## Results

**Fig 1:**
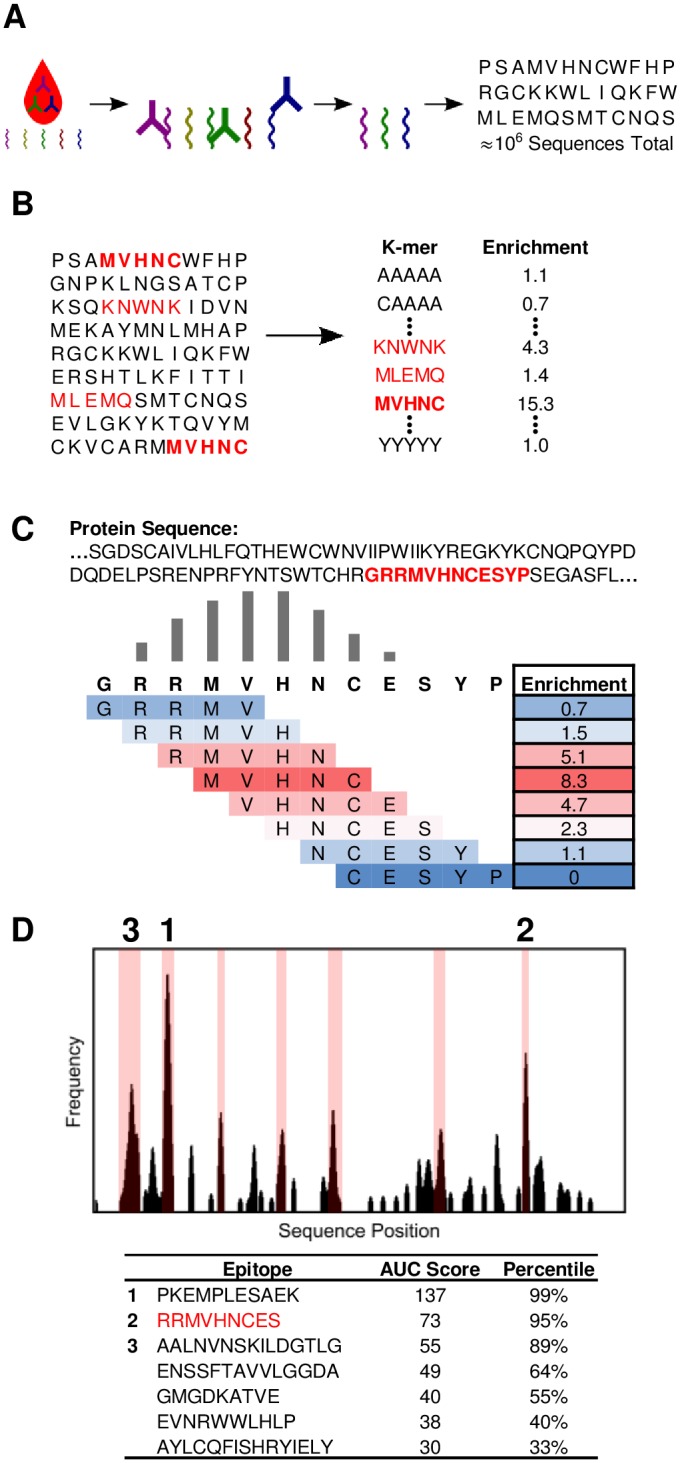
K-TOPE determines epitopes by tiling proteins into k-mers. (A) The input to the algorithm is a dataset of approximately 10^6^ peptides that were bound by serum antibodies. (B) All 5-mers are evaluated for their enrichment in the list of peptides. (C) A portion of a protein sequence is tiled into 5-mers which are weighted by their enrichment. This determines a “frequency” value for each position in the sequence. (D) The frequency value for each position in a protein sequence is plotted as a histogram. Possible epitopes are highlighted in pink on the graph. Epitope sequences, area under the curve (AUC) scores, and significance percentiles are displayed.

To enable the prediction of protein epitopes bound by serum antibodies, we developed a method that uses a database of antibody-binding peptides to predict epitopes in known protein sequences (Fig 1). First, we selected peptides binding to an individual antibody repertoire within a specimen (serum or plasma) from a bacterial display peptide library with 10^10^ random 12-mer members. Then, we identified antibody-binding peptide sequences using NGS. To allow for the manipulation of 20^5^ (3.2 million) k-mers rather than full-length peptides, we processed peptides into subsequences and evaluated the enrichments of all k-mers of length 5 [29]. We chose 5-mers because virtually all 5-mers were found in the peptide library at least once (S1 Text). Next, K-TOPE tiled candidate antigen sequences, such as from a proteome, into overlapping k-mers. K-TOPE used the enrichment values for these k-mers to construct an enrichment histogram across the length of each protein sequence. The frequency value at each sequence position in the histogram was proportional to the enrichment of k-mers that included that position. Specifically, for all k-mers overlapping a position, we summed the log base 2 of the k-mer enrichment. Thus, higher frequency values at a position in a protein sequence corresponded to a greater probability that a position was included in an epitope. All subsequences between two minima in the histogram with non-zero frequency values were considered “potential epitopes”. These potential epitopes were scored based on the area under the curve (AUC). Next, potential epitopes were assigned an “epitope percentile” based on the rank of the epitope’s AUC score in a list of AUC scores generated by analyzing random proteins. Finally, a threshold was set on the epitope percentile to determine whether an individual epitope was considered bound or simply noise. For this study an epitope percentile threshold of 95% was used, which corresponds to a p-value of 0.05. The prevalence of each epitope was calculated as the proportion of specimens that bound the epitope.

**Fig 2:**
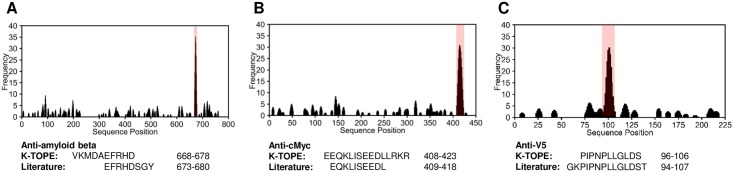
K-TOPE found epitopes for antibodies with known specificity spiked into serum. Histograms for antibodies with known specificity against amyloid beta (P05067), cMyc (P01106), and V5 (P11207) had prominent epitopes with epitope percentiles > 99.9% (in pink). (A) K-TOPE analysis of amyloid beta determined the epitope VKMDAEFRHD (668–678). This antibody was raised to whole protein and is known from literature to have a conformation-specific discontinuous epitope that maps to segments EFRHDSGY (673–680) and ED (692–693). (B) K-TOPE analysis of cMyc determined the epitope EEQKLISEEDLLRKR (408–422). This antibody was raised to AEEQKLISEEDLLRKRRE (407–424). (C) K-TOPE analysis of V5 determined the epitope PIPNPLLGLDS (96–106). The antibody was raised to GKPIPNPLLGLDST (94–107).

To assess the utility of K-TOPE, we first determined epitopes for monoclonal and polyclonal antibodies that bind specific, well-defined epitopes in cMyc, V5, and amyloid beta. We spiked these antibodies into serum at a final concentration of 25 nM and then selected and identified binding peptides. K-TOPE predicted epitopes that had greater than 60% overlap with the previously reported epitopes of these antibodies (Fig 2). Importantly, the enrichment histograms generated by antibodies spiked into background serum or buffer were nearly identical (S1 Fig), suggesting that the noisy serum environment minimally affected epitope identification.

**Fig 3:**
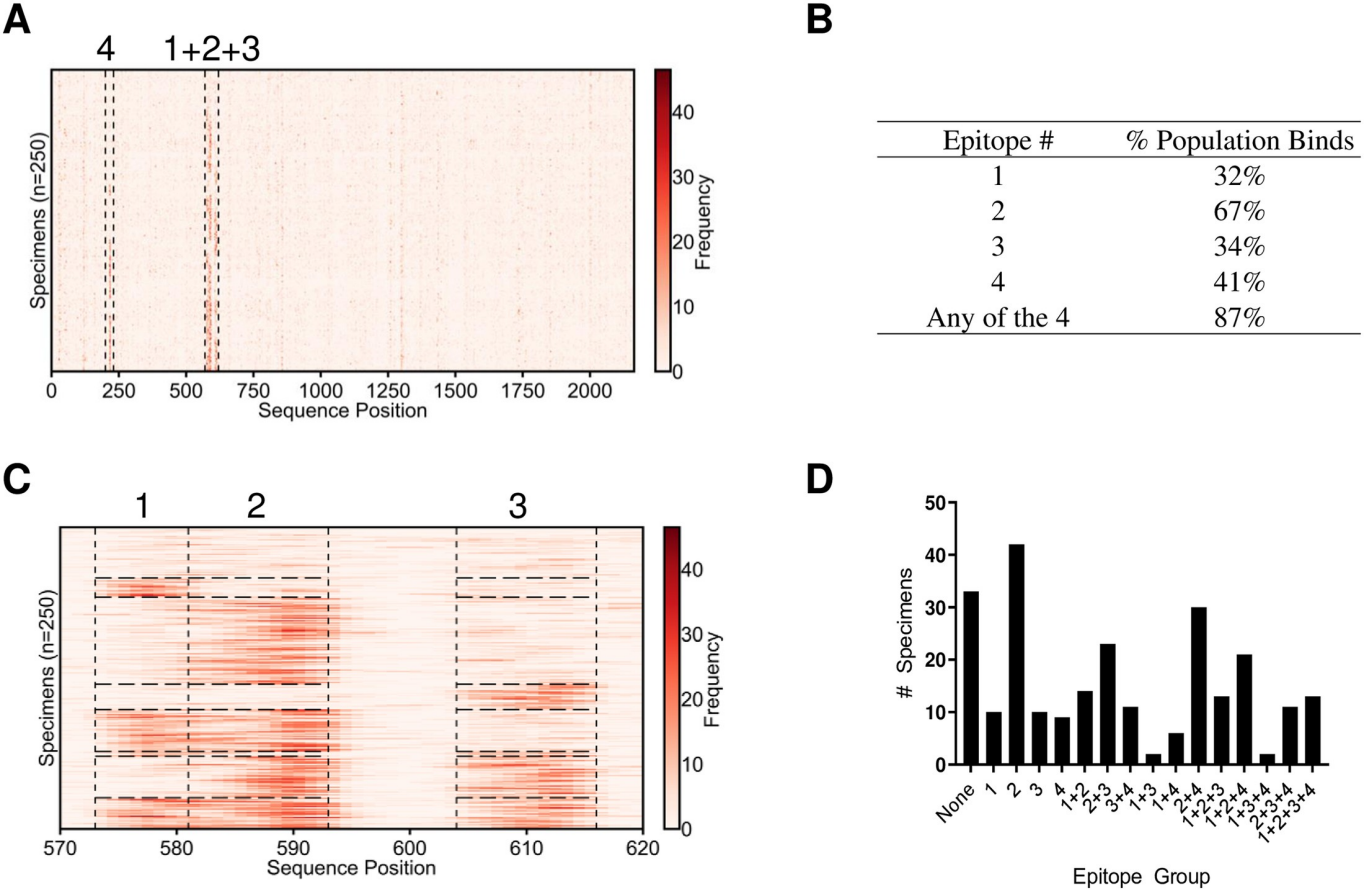
K-TOPE predicted four epitopes in the *Rhinovirus A* genome polyprotein. (A) K-TOPE was applied to the *Rhinovirus A* genome polyprotein (P07210) for 250 specimens. Histograms for all specimens are shown as rows in a heat map. The specimens have been clustered such that specimens that bind the same epitopes are adjacent. Regions that contain epitopes are outlined by dotted lines. (B) A table of the percentage of the population that bound each epitope. (C) The region from positions 570–620 is divided into 3 sections that correspond to distinct epitopes. These epitopes are consensus epitopes which were present in >30% of the 250 specimens. (D) Bar graph showing membership in different epitope groups. For example, a specimen that binds epitopes 2 and 3 will belong to epitope group “2+3”. In this population, 87% of the specimens bound at least one of the consensus epitopes. The sequences of the epitopes were 1: QNPVENYI, 2: DSVLEVLVVPN, 3: APALDAAETGHT, and 4: NHTHPGEQG.

To predict “public epitopes” conserved across many individuals, epitopes were predicted for each specimen individually and then clustered. Although many private epitopes were predicted for each specimen in this process, we focused on the far smaller set of public epitopes to facilitate comparison with previous literature. Given the ubiquity of exposure to the upper respiratory pathogen *Rhinovirus A*, we validated the approach by predicting epitopes within its genome polyprotein. The true epitope percentile indicative of antibody binding and the true prevalence suggesting clinical relevance vary by antibody and the determination of the optimal values of these parameters would require additional experimental validation. However, for the purposes of this study, the epitope percentile threshold (S2 Fig) and prevalence (S3 Fig) were varied and an epitope percentile threshold of 95% and a prevalence of 30% were chosen. These values were chosen to ensure that the total number of epitopes predicted was of order one with the goal of decreasing the inclusion of false positives. Using a unique set of 250 serum specimens, we predicted four epitopes within *Rhinovirus A* that were targeted by 30% or more of the specimens (Fig 3A). Of the 250 specimens, 87% exhibited binding to at least one of these consensus epitopes (Fig 3B). Three of these epitopes were located within positions 570–620 (Fig 3C), in the antigenic attachment region of VP1. A fourth epitope within the VP2 region of the *Rhinovirus A* genome polyprotein was targeted by 43% of the population.

To assess trends in the population, each specimen was assigned into one of 16 groups based on which of the four *Rhinovirus A* epitopes were bound (Fig 3D). Notably, epitope binding was not independent, since 5 of the 16 groups of specimens were at least 50% larger than expected and the group targeting epitopes ‘1+3’ was 60% smaller than expected (S1 Table). The average age of the subset of specimens of known age (n = 138) was 35 years. However, the specimens targeting 3 or more epitopes had an average age of 17, which was approximately 50% lower than the average age of 35 and the epitope group targeting none of the epitopes had an average age of 52, which was approximately 50% higher than average age of the population (S2 Table). Thus, people who targeted fewer *Rhinovirus A* epitopes tended to be older.

To establish whether these *Rhinovirus A* epitopes were strain-specific, we predicted epitopes using KTOPE for 43 different *Enterovirus* strains (S3 Table). The epitopes predicted for these *Enterovirus* strains were similar to the 4 epitopes predicted in Fig 3, as illustrated by bands in the heat map showing the positions of each epitope (S4 Fig) Epitopes 1, 2, and 4 from Fig 3 were only found in *Rhinovirus*, whereas epitope 3 from Fig 3 was found in many *Enterovirus* strains. These results suggest that the epitopes predicted for *Rhinovirus A* may be relevant to multiple other *Enterovirus* strains.

Next, we investigated the utility of using K-TOPE to predict epitopes within a set of 2,908 proteins from 400 viral taxa with human tropism. This approach yielded 29 epitopes that were bound by at least 30% of all specimens (Table 1). Some of these epitopes have been reported previously [6,33–35]. Thus, a modest number of prominent linear viral epitopes were bound by >30% of the specimens analyzed. A common antigen identified from this analysis was Epstein-Barr nuclear antigen 1 (EBNA1) from Epstein-Barr virus (EBV), which is expressed in EBV-infected cells [36]. Additionally, the epitopes predicted for the enterovirus genus were consistent with the epitopes predicted for *Rhinovirus* A, which is a species in that genus (Fig 3). Several of the epitopes were likely due to false discovery (e.g., Mayaro virus and Lyssavirus), since these viruses are uncommon in a general population. There is an intrinsic lower limit on false positives since antibodies only bind 5–6 amino acids, which is not enough information to uniquely specify a protein subsequence. This limitation is especially pronounced among evolutionarily related proteins in closely related species. To decrease the incidence of false positives, K-TOPE should only be used to analyze biologically relevant proteins. Ultimately, the epitopes predicted by K-TOPE require experimental validation to eliminate spurious results.

**Table 1:** A collection of 29 viral epitopes to which >30% of 250 specimens bound.

Epitope	Protein	Taxon	Accession	Prevalence
DSVLNEVLVVPN	Genome polyprotein	Enterovirus	P07210	0.668
PALTAAETG	Genome polyprotein	Enterovirus	Q66575	0.588
GRRPFFHPV	Epstein-Barr nuclear antigen 1	Epstein-Barr virus (strain GD1)	Q1HVF7	0.524
AGAGGGAGA	Epstein-Barr nuclear antigen 1	Epstein-Barr virus (strain GD1)	Q1HVF7	0.516
KYTHPGEA	Genome polyprotein	Enterovirus	Q82122	0.492
VRRPFFSD	Protein UL84	Human cytomegalovirus	P16727	0.452
NPVERYVDE	Genome polyprotein	Enterovirus	Q82122	0.428
MVVPEFK	DNA-binding protein	Human mastadenovirus C	P03265	0.428
EVKLPHWTPT	Glycoprotein 42	Epstein-Barr virus (strain GD1)	P03205	0.42
KPQPEKPK	Structural polyprotein	Mayaro virus	Q8QZ72	0.416
GGAGAGGAGAGGG	Epstein-Barr nuclear antigen 1	Epstein-Barr virus (strain GD1)	P03211	0.412
ININRPLE	Large structural protein	Lyssavirus	Q9QSP0	0.412
RPSCIGCKG	Epstein-Barr nuclear antigen 1	Epstein-Barr virus (strain GD1)	P03211	0.404
GAGAGAGGG	Packaging protein UL32	Simplexvirus	P89455	0.376
LEEVIVEKTK	Genome polyprotein	Enterovirus	Q82081	0.352
KHTHPGI	Replication origin-binding protein	Human herpesvirus 3	P09299	0.352
AETGHTNKI	Genome polyprotein	Enterovirus	Q82122	0.344
YVFPHWITK	Envelope glycoprotein gp63	Primate T-lymphotropic virus 3	Q0R5Q9	0.34
KTTNTTTNT	Immediate-early protein 2	Roseolovirus	Q9QJ16	0.34
MAADKPTL	Genome polyprotein	Murray Valley encephalitis virus	P05769	0.34
SFIVPEFA	Virion membrane protein A16	Orthopoxvirus	P16710	0.332
LVLPHWYMA	Cytoplasmic envelopment protein 1	Simplexvirus	P89430	0.328
YVDDMLNDI	Large tegument protein deneddylase	Human herpesvirus 6A (strain Uganda-1102)	P52340	0.328
SSGPKHTQKV	Genome polyprotein	Enterovirus	P03303	0.324
PVPEFQA	Non-structural polyprotein	Semliki forest virus	P08411	0.316
VPVTPNIAI	Genome polyprotein	Hepatitis C virus	Q68749	0.304
LHRPALTA	Minor capsid protein L2	Human papillomavirus type 34	P36758	0.304
EHILNRPTG	RNA-directed RNA polymerase L	Crimean-Congo hemorrhagic fever orthonairovirus	Q6TQR6	0.304
GEFIGSE	Shutoff alkaline exonuclease	Human herpesvirus 8	Q2HR95	0.3

K-TOPE was used to analyze 2,908 proteins from viruses with human tropism. This search demonstrated that only a few prominent linear viral epitopes were bound by a large proportion of the population.

We performed a similar analysis for the proteomes of the genera *Streptococcus* and *Staphylococcus*, which are common bacterial human pathogens with 2,976 and 3,071 proteins in their respective proteomes. K-TOPE was used with each of these proteomes to determine epitopes bound by >30% of a population of 250 specimens, yielding 9 epitopes for *Streptococcus* and 13 epitopes for *Staphylococcus* (Table 2). The epitope LIPEFIG(R) in ATP-dependent Clp protease ATP-binding subunit ClpX was the most prevalent *Streptococcus* epitope and second most prevalent *Staphylococcus* epitope. Therefore, K-TOPE could not determine which genus generated this epitope. The most prevalent *Staphylococcus* epitope was PTHYVPEFKGS from extracellular matrix protein-binding protein emp, which is a known virulence factor [37]. For *Streptococcus*, the second most prevalent epitope was GQKMDDMLNS from the highly antigenic Streptolysin O protein [38]. This epitope falls within a 70 amino acid range in Streptolysin O that is known to bind antibodies [39]. The sequence “DKP” was present in 5/9 *Streptococcus* epitopes and the sequence “PEFXG” was present in 6/13 *Staphylococcus* epitopes (Table 2). Therefore, there are multiple candidate antigens that may correspond to these highly enriched sequences.

**Table 2:** Epitopes in the proteomes of the genera *Staphylococcus* and *Streptococcus* which were bound by >30% of 250 specimens.

Epitope	Protein	Accession	Prevalence
***Streptococcus***			
LIPEFIGR	ATP-dependent Clp protease ATP-binding subunit ClpX	P63793	0.512
GQKMDDMLNS	Streptolysin O	Q5XE40	0.436
QIPALDKPL	FMN-dependent NADH-azoreductase	A4W2Z7	0.416
IADKPILD	UPF0154 protein SSU05_1707	A4VX34	0.392
TVADKPVA	Phenylalanine--tRNA ligase beta subunit	Q5XCX3	0.360
RTPDKPT	Agglutinin receptor	P16952	0.324
VVPNIWR	Putative 2-dehydropantoate 2-reductase	P65666	0.320
LLNRPIHD	CCA-adding enzyme	Q5M153	0.320
TLADKPEF	Autolysin	P06653	0.308
***Staphylococcus***			
PTHYVPEFKGS	Extracellular matrix protein-binding protein emp	Q2FIK4	0.572
LIPEFIG	ATP-dependent Clp protease ATP-binding subunit ClpX	B9DNC0	0.508
NKPEFSGAT	3-isopropylmalate dehydratase small subunit	Q4L7U3	0.436
NKNNKNNKN	Translation initiation factor IF-2	Q4L5X1	0.372
KLGNIVPEYK	Extracellular matrix protein-binding protein emp	P0C6P1	0.360
KLCRICFRE	30S ribosomal protein S14 type Z	Q5HM12	0.352
DFLNRPVD	Proline--tRNA ligase	Q4L5W5	0.348
EKNNNNNNNNS	Alkaline shock protein 23	Q4L860	0.320
GVVPNISR	UvrABC system protein A	Q5HHQ9	0.312
LIPEFNQV	Homoserine kinase	Q8CSQ2	0.308
SPEFLGSQ	Undecaprenyl-diphosphatase	B9DK59	0.308
VGINRPTY	Putative glycosyltransferase TagX	O05154	0.308
VIPEFNND	Peptide chain release factor 2	Q4L4H9	0.300

K-TOPE was used to analyze 2,976 proteins from *Streptococcus* and 3,071 proteins from *Staphylococcus*.

**Fig 4:**
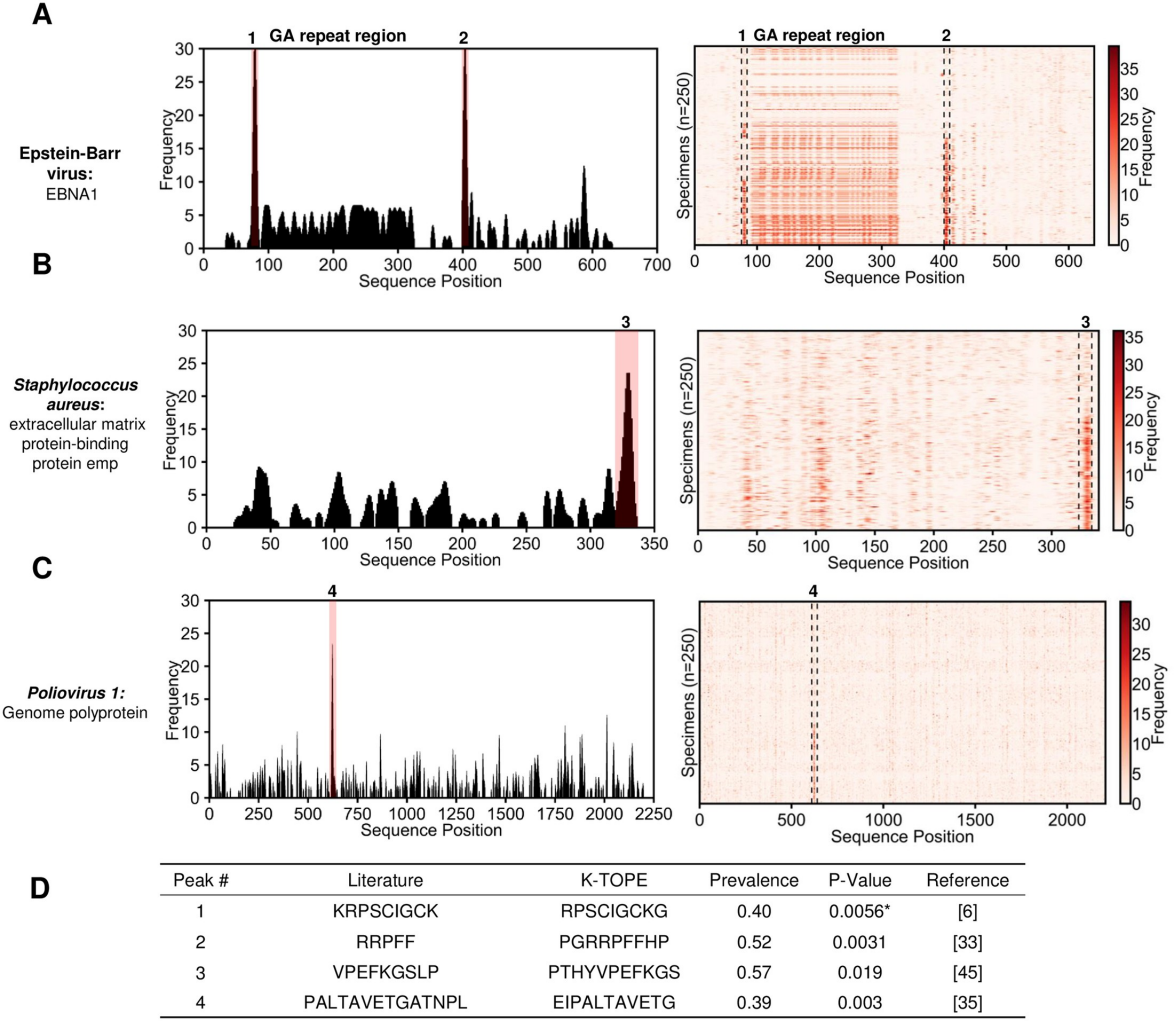
Epitopes predicted through proteome searches were validated using literature-reported epitopes. In (A), (B), and (C), histograms are shown for typical individual specimens (epitopes with percentiles > 99.7% are highlighted in pink). To the right of the histogram is a heat map for 250 specimens. For (A), there is a region of antigenic GA-repeats from positions 100–350. The table in (D) provides the statistical significance of agreement between literature epitopes and K-TOPE epitopes for the labeled peaks in (A), (B), and (C). The UniProt accessions used for this analysis were P03211 for EBNA1, Q8NXI8 for extracellular matrix protein-binding protein emp, and P03300 for *Poliovirus 1* Genome Polyprotein. Statistical tests where epitopes with >50% GA content were removed are denoted by an asterisk “*”. All predicted epitopes had p-values below 0.05.

We searched IEDB ([40]) to determine which of the 51 viral and bacterial epitopes predicted by KTOPE were previously identified (S4 Table). Twelve of the 51 epitopes were similar to epitopes found in prior studies ([6,23,33,35,39,41–45]). However, 30 of the epitopes were in proteins with no reported epitopes, and 3 epitopes were in organisms with no reported epitopes. Only 6 of the epitopes were in well-characterized proteins but were not found in the literature, suggesting that these epitopes were false positives or novel epitopes. Additionally, only two bacterial epitopes were in previously described proteins, suggesting that the remainder of the bacterial proteins were false positives or novel antigens. Literature validation is shown in Fig 4 for the viral proteins EBNA1 from EBV and the *Poliovirus 1* genome polyprotein, as well as the bacterial protein Extracellular matrix protein-binding protein emp from *Staphylococcus*. In these cases, K-TOPE found prominent peaks in the histograms that corresponded to reported epitopes (Fig 4) [6,33,35,45]. Additionally, K-TOPE identified an immunogenic region of GA-repeats from positions 100–350 in the analysis of EBNA1 [23]. We used a nonparametric statistical test to assign significance to the overlap between K-TOPE epitopes and known epitopes. Using this method, all epitopes evaluated using K-TOPE had P-values below 0.05 (Fig 4C).

**Fig 5:**
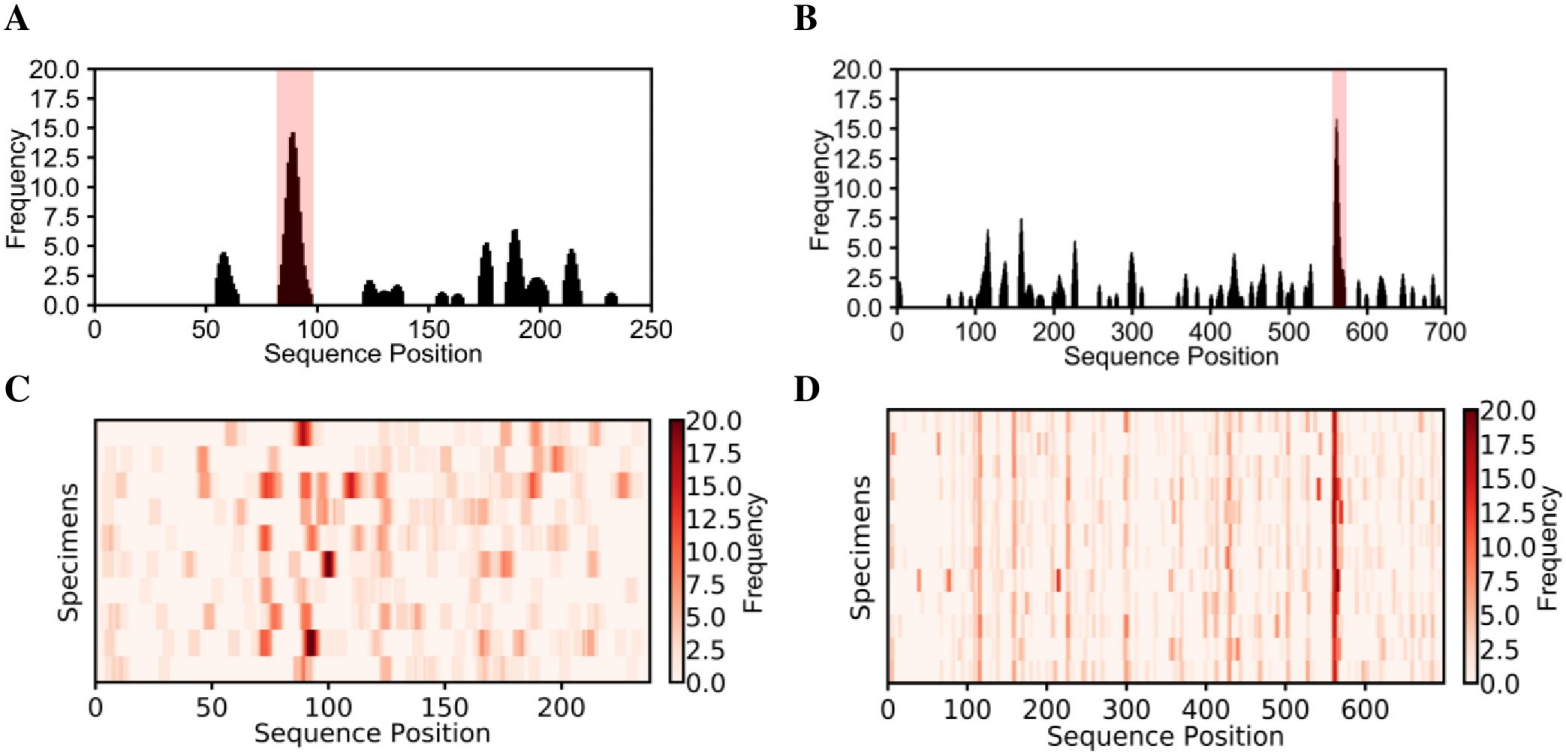
K-TOPE predicted epitopes for glycoprotein G1 using HSV1 specimens and for glycoprotein G2 using HSV2 specimens. For glycoprotein G1, a representative histogram for a single specimen is shown in (A) and a heat map for all HSV1 specimens is shown in (C). For glycoprotein G2, a representative histogram for a single specimen is shown in (B) and a heat map for all HSV2 specimens is shown in (D). There was a single epitope predicted for each protein. Epitopes with percentiles > 97% are highlighted in pink.

To predict HSV species-specific epitopes, we analyzed 12 HSV2 specimens and 10 HSV1 specimens. Since these viruses share many of the same proteins in their proteomes [11], HSV1 specimens were appropriate controls for HSV2 specimens and vice-versa. To begin, we predicted species-specific epitopes in glycoprotein G, which is a protein that varies significantly between the two species (Fig 5) [46]. There was a single HSV1 epitope, PMPSIGLEE, bound by 40% of HSV1 specimens and a single HSV2 epitope, GGPEEFEGAGD, bound by all HSV2 specimens. This HSV2-specific epitope aligned well with previous epitopes found for glycoprotein G2 [13,47,48] (Table 3). Also, this epitope has been validated as an HSV2-specific diagnostic [49,50]. The HSV1-specific epitope was also similar to the previously reported epitope DHTPPMPSIGLE [18]. Interestingly, the two HSV-specific epitopes terminated in an identical 7-mer sequence EGAGDGE (PMPSIGLEEEEEE**EGAGDGE** and GGPEEF**EGAGDGE**) [47]. This suggests that the regions containing these epitopes may be evolutionarily or structurally related targets of the immune system.

**Table 3:** Alignment of an HSV2-specific glycoprotein G2 epitope with previously reported epitopes.

Peptides																													Reference
								G	G	**P**	**E**	**E**	**F**	**E**	**G**	**A**	**G**	**D**											K-TOPE
										**P**	**E**	**E**	**F**	**E**	**G**	**A**	**G**	**D**	G	E	P	P	E	D	D	D	S	G	[13]
	P	P	P	P	E	H	R	G	G	**P**	**E**	**E**	**F**	**E**	**G**	**A**	**G**	**D**	G	E	P	P	E						[47]
A	P	P	P	P	E	H	R	G	G	**P**	**E**	**E**	**F**	**E**	**G**	**A**	**G**	**D**	G										[48]

To predict candidate HSV species-specific epitopes, we analyzed the HSV1 and HSV2 proteomes. We predicted 30 HSV2-specific epitopes that were 100% specific with prevalence > 30% (Table 4). Notably, 11 of these epitopes were bound by all HSV2 specimens. K-TOPE predicted a glycoprotein C epitope PRTTPTPPQ with 83% prevalence which was contained in a previously identified epitope RNASA**PRTTPTPPQ**PRKATK [18]. In contrast to the numerous HSV2-specific epitopes, only 4 HSV1-specific epitopes were predicted, and the highest prevalence achieved was only 40% (Table 5). One of these epitopes, RIRLPHI, overlapped with the previously identified epitope HRRTRKAPK**RIRLPHI**R [51] in the well-described antigen glycoprotein D [17]. One possible explanation for the discovery of fewer HSV1-specific epitopes is that the HSV2 specimens had high IgM levels, whereas the HSV1 specimens had high IgG levels. Since high IgM levels occur with severe recurrent herpes infections [52], we would expect the high IgM HSV2 sera to yield more epitopes.

**Table 4:** HSV2-specific epitopes were predicted.

Epitope	Protein	Accession	Prevalence
GGPEEFEGAGD	Envelope glycoprotein G	P13290	1
PLYARTTPAKF	Tegument protein UL47	P89467	1
VDSQRLTPGGSVS	Tegument protein UL21	P89444	1
KARKKGTSAL	Envelope glycoprotein B	P08666	1
TPLRYACVL	Tegument protein UL47	P89467	1
ANSPWAPVL	mRNA export factor	P28276	1
RYSPLHN	Envelope glycoprotein B	P08666	1
EAMLNDAR	Large tegument protein deneddylase	P89459	1
QRLTPH	Large tegument protein deneddylase	P89459	1
LRYTPAGEV	Envelope glycoprotein H	P89445	1
RTPSMR	Major viral transcription factor ICP4 homolog	P90493	1
LATNNA	Small capsomere-interacting protein	P89458	0.917
LRTNNL	Ribonucleoside-diphosphate reductase small subunit	P69521	0.917
PRTTPTPPQ	Envelope glycoprotein C	Q89730	0.833
HRLYAVVA	Inner tegument protein	P89460	0.833
PSTPAMLNLG	Ribonucleoside-diphosphate reductase large subunit	P89462	0.667
VTKHTALCAR	Large tegument protein deneddylase	P89459	0.583
TRDYAGL	Envelope glycoprotein I	P13291	0.583
RLTVAQ	Envelope glycoprotein I	P13291	0.583
RSLGIA	Protein UL20	P89443	0.583
IRDLARTFA	Thymidine kinase	P89446	0.5
DITAKHRCL	Major capsid protein	P89442	0.5
ETPAQPPRY	Capsid scaffolding protein	P89449	0.5
VSGITPTQ	Tripartite terminase subunit 1	P89451	0.5
HEELYYGPVS	Tegument protein VP22	P89468	0.417
IQDLAYAIV	Ribonucleoside-diphosphate reductase large subunit	P89462	0.417
GPAQRHTY	DNA polymerase catalytic subunit	P89453	0.417
YFEEYAYS	Envelope glycoprotein B	P08666	0.417
LDDFDL	Tegument protein VP16	P68336	0.417
AARLIDALYAEFLGG	Envelope glycoprotein H	P89445	0.333

A total of 30 epitopes were predicted that were 100% specific against HSV1.

**Table 5:** HSV1-specific epitopes were predicted.

Epitope	Protein	Accession	Prevalence
RIRLPHI	Envelope glycoprotein D	Q69091	0.4
PMPSIGLEE	Envelope glycoprotein G	P06484	0.4
CAAFVNDYSLV	Major capsid protein	P06491	0.3
EMADTFLDT	ICP47 protein	P03170	0.3

Only 4 epitopes were predicted that were 100% specific against HSV2.

We sought to determine whether the HSV2-specific epitopes were contained in proteins that differed between the HSV species [46]. We determined 8 HSV2-specific epitopes with sequences that were contained in both HSV proteomes (S5 Table). Our analysis suggested that these epitopes were only targeted by HSV2 specimens, despite their presence in the HSV1 proteome. Thus, even sequences that are conserved between species could serve as species-specific targets.

Since HSV1 is common in the general population, we were interested in identifying similarities in the epitopes predicted using the HSV1 specimens (Table 5) and the 250 specimens. Through this analysis we predicted 30 epitopes that were found in at least 10% of the 250 specimens (S6 Table). Notably, epitope 1 (FVLPHWYM) contains the 3-mer LPH which is also in the first epitope of the HSV1-specific epitopes, RIRLPHI (Table 5). Additionally, epitope 21 (PMPSLTA) contains the 4-mer PMPS which is also in epitope 2 of the HSV1-specific epitopes, PMPSIGLEE. A nearly exact match was found for epitope 18 (AAFVNDYS), which is highly similar to epitope 3 in the HSV1-specific epitope list, CAAFVNDYSLV. Thus, 3 of the 4 epitopes discovered from an HSV1-infected population had similarities to epitopes found using a general population. Additionally, 3 of the 4 antigens predicted from analyzing the 250 specimens were also predicted using the HSV1-infected specimens (major capsid protein, envelope glycoprotein G, and envelope glycoprotein D). Fewer epitopes were identified for the HSV1-infected specimens than the 250 specimens due to the group size disparity (10 specimens vs 250 specimens) and since the epitopes predicted from the HSV1-infected specimens used HSV2-infected specimens as controls. Although the prevalence of HSV1 is nearly 50% [9], we did not find epitopes with a prevalence this high, likely because there are a variety of HSV1 epitopes that collectively indicate a prevalence of 50%. Hence, individual epitopes may only have prevalence values of 10–30%.

## Discussion

Here, we present a generalizable methodology for predicting epitopes within candidate immunogenic proteins. By tiling proteins into k-mers and evaluating those k-mers in a database of antibody-binding peptides, we determined epitopes for individuals and a population. Importantly, we have demonstrated that K-TOPE can predict disease-specific epitopes and antigens. One of the main features of this approach is that it combines k-mers to determine composite epitopes that may not explicitly exist in the peptide dataset. Another important element is using an antigen sequence to predict epitopes, thereby surmounting the 7 amino acid requirement for successful antigen identification [30].

The K-TOPE approach to epitope mapping differs from reported methods in several important ways. While proteome-derived peptide libraries have been used to predict disease-specific epitopes [33,53], these methods lack the flexibility to examine multiple proteomes. For instance, separate libraries would be required to analyze both HSV1 and HSV2. Even a library that contains peptides spanning all viral proteomes cannot easily be extended to much larger bacterial or parasitic proteomes [24]. A disadvantage of microarrays is that they have far lower 5-mer coverage (~27% [32]), than surface display (~100%) which could limit the application of k-mer approaches. Other algorithms have been developed that predict binding motifs in peptide datasets, but they lack the integrated capability to connect motifs to protein antigens [54,55]. Also, the direct method of aligning peptides to sequences becomes computationally infeasible with a large number of peptides and candidate antigens [56].

The heterogeneity of experimental approaches complicates the validation of putative epitopes and their associated antigens. The Immune Epitope Database (IEDB) has an all-inclusive representation of information [57], which may not reflect important distinctions in experimental platforms, specimens, and data analysis techniques. For instance, there are likely numerous false positive epitopes for highly studied organisms and few identified epitopes for poorly studied organisms. Also, there is a lack of quantitative data reported for epitopes [58], such as the proportion of a given population that binds an epitope. To address this lack of information, we first used K-TOPE to analyze specimens for responses to common pathogens in a general population. This allows newly predicted “public epitopes” to be benchmarked by nearly any set of serum specimens. We required that a proportion of the population bind an epitope to reduce false positives. Although analysis of the variation in private epitopes could be valuable for understanding the variation in immune responses, it would complicate validation. We determined public epitopes in *Rhinovirus A* and showed that people who targeted fewer *Rhinovirus A* epitopes tended to be older, perhaps due to immunosenescence [59], reduced pathogen exposure, or a lower incidence of rhinovirus infections [60]. With a diverse group of specimens, it was possible to confirm that the RRPFF epitope in EBV’s protein EBNA1 is a very commonly targeted epitope [33]. Since the specimens used to determine public epitopes were not assayed for responses to pathogens, acute and chronic infections could not be readily distinguished from prior infections. These public epitopes could be further validated using specimens with acute infections or using longitudinal studies to determine if these epitopes appear upon vaccination [61]. We did not find epitopes corresponding to measles or rubella vaccination, which is consistent with a recent study that comprehensively predicted viral epitopes [62]. This implies that for these viruses, high titer antibodies targeting linear epitopes may not be present. For HSV1 and HSV2, we determined whether an epitope was specific by analyzing specimens infected by both virus species. Unexpectedly, we demonstrated that even epitopes present in the conserved regions of both species’ proteomes could be species-specific. The difference in binding was likely due to differences in the structure and post-translational modifications of the proteins. For the HSV analysis, we validated epitopes using previous studies, however, it was difficult to know *a priori* whether a non-validated epitope was novel or spurious. In general, since studies use different specimens, experiments, and computational analyses, it is unlikely for the epitopes of two studies to completely coincide.

K-TOPE provides a new tool for identifying diagnostic biomarkers, vaccine components, and candidate therapeutic targets. This approach could be used in the iterative process of designing a vaccine, since it would be useful to know which epitopes are elicited in a population by vaccination. Vaccine formulation could be altered to maximize the percentage of the population that targets epitopes associated with a positive disease outcome [2]. K-TOPE could also enable the development of diagnostics that assign disease based on the presence of epitopes. Since this method only involves a single experimental screen, in principle multiple diseases could be simultaneously diagnosed [63]. By searching for consensus epitopes in a disease group that are absent in a control group, K-TOPE can discover disease-specific epitopes. For an autoimmune disease, the entire human proteome could be analyzed to determine autoantigen epitopes [33]. Similarly, using clinical histories of viral infection, K-TOPE can analyze the proteomes of suspected pathogens to link epitopes to infections [24]. With specimens that have HLA information, it could be possible to detect a correlation between HLA type and bound epitopes [64]. This could have implications for how we determine genetic predisposition to immunological disease.

There are important limitations to the conditions in which this approach could be successful. First, this approach is currently limited to the prediction of linear epitopes. However, since 85% of epitopes have at least one linear stretch of five amino acids [22], conformational epitopes with linear segments may be represented in the datasets. We chose to focus on linear epitopes since methods that predict conformational epitopes often require 3D protein structures, which are scarce relative to the number of protein sequences. This report focuses on epitopes from common pathogens which are high-titer, but it could be difficult to detect rare antibody epitopes. Methods that selectively deplete out high-titer antibodies could prove effective for probing rare antibodies [65]. Another limitation is that protein sequences tend to have a large degree of conservation and redundancy [66], as demonstrated by the false positives found in the viral epitope search. Thus, even for analyses of non-immunogenic proteomes, false positives will occur due to evolutionary or coincidental sequence overlap with immunogenic proteomes. The issue of false positives can be partially allayed by deliberately choosing the set of investigated proteins, such that all proteins are plausible candidate antigens. Sequence conservation was demonstrated with the Enterovirus epitope PALTAVETGATNPL [35], as well as with the *Human herpesvirus 6A* epitope YVDDMLNDI (Table 1) which shares the k-mer “DDMLN” with the *Streptococcus* epitope GQKMDDMLNS (Table 2). Generally, if an epitope sequence is present identically in multiple antigens, all candidate antigens should be considered equally plausible without further biological, epidemiological, or experimental information. It is important to note that one of the purposes of K-TOPE is to reduce thousands of candidate proteins to a small set of proteins that can be experimentally validated.

In summary, the present approach enables the discovery of epitopes within the proteomes of any organism whose sequence is deposited into the protein database. The challenge of associating epitopes with antigens can be surmounted by transforming sets of antibody-binding peptides to k-mers and tiling proteins of interest. Advancements upon this paradigm may enable comprehensive immunological evaluations from serum and other biological tissues.

## Materials and methods

### Strains and reagents

*E*. *coli* strain MC1061 was used with surface display vector pB33eCPX for all library screening experiments. Protein A/G magnetic beads were from Thermo Scientific Pierce. Antibodies with known specificity included C3956 rabbit anti-c-Myc polyclonal antibody (Sigma), anti-beta amyloid 1–42 antibody [mOC31]—conformation-specific (ab201059) (Abcam), and rabbit V8137 Anti-V5 polyclonal antibody (Sigma). Antibodies were spiked into healthy donor serum at a concentration of 25 nM. All sera (n = 273) were obtained as deidentified specimens from biobanks according to institutional guidelines (Table 6), (Biosafety authorization numbers #201417, #201713), and handled according to CDC-recommended BSL2 guidelines.

**Table 6:** Serum sources.

Received From	# Specimens	Ages
UCLA	26	67 ± 10.5
UCSF	32	62 ± 7.2
Mayo Clinic	9	N/A
National Institute for Health and Welfare (Helsinki, Finland)	90	10 ± 5
NIH	40	N/A
Santa Barbara Cottage Hospital	12	N/A
Johns Hopkins University	15	30 ± 11.8
Max Delbrück Center for Molecular Medicine	26	N/A
BioreclamationIVT (HSV2)	12	N/A
Discovery Life Sciences (HSV1)	10	N/A
UCSB (mAb study)	1	N/A

Specimens that were provided without age information are noted by “N/A” under “Ages”. Of the 90 specimens from the National Institute for Health and Welfare (Helsinki, Finland), 25 specimens were provided without age information. The average age is given with the standard deviation.

### Bacterial peptide display and sequencing

The bacterial peptide display screening protocol was carried out as previously described [29,67]. Briefly, an *E*. *coli* library displaying approximately 8 billion different 12-mer peptides was combined with 1:100 diluted serum. We used magnetic selection with Protein A/G beads to isolate bacterial cells with bound antibodies. Then, we confirmed that this isolated fraction of bacteria bound antibodies using flow cytometry. Amplicons were prepared from the isolated fraction for sequencing using the Illumina NextSeq.

### Protein databases

Protein sequences were obtained from UniProt or by using the Biopython module [68]. Accessions for proteins are noted in figures and figure captions. For the epitope validation in Fig 4, accessions were chosen that reference the most highly annotated version of the proteins identified in Tables 1 and 2. The list of random proteins used for statistical analysis was obtained through a UniProt search of “reviewed:yes”. The list of *Enterovirus* strains was obtained from a UniProt search of “enterovirus NOT organism:"homo sapiens" AND reviewed:yes”. The viral proteome search used a Uniref search of “uniprot:(host:"homo sapiens" reviewed:yes fragment:no) AND identity:0.9” and yielded 2,908 proteins. The *Staphylococcus* proteome search used a Uniref search of “uniprot:(taxonomy:"Staphylococcus [1279]" fragment:no reviewed:yes) AND identity:0.9” and yielded 3,071 proteins. The *Streptococcus* proteome search used a Uniref search of “uniprot:(taxonomy:"Streptococcus [1301]" fragment:no reviewed:yes) AND identity:0.9” and yielded 2,976 proteins. HSV analysis used a UniProt search of “reviewed:yes AND organism:"Human herpesvirus 1 (strain 17) (HHV-1) (Human herpes simplex virus 1) [10299]" AND proteome:up000009294” for HSV1, yielding 73 proteins and a Uniprot search of “reviewed:yes AND organism:"Human herpesvirus 2 (strain HG52) (HHV-2) (Human herpes simplex virus 2) [10315]" AND proteome:up000001874” for HSV2, yielding 72 proteins.

### Literature epitopes

For EBNA1, it was noted that RRPFF antibodies were found in the serum of healthy individuals [33]. KRPSCIGCK was noted as an EBNA1 epitope that was preferentially targeted by pre-eclamptic women, but was also targeted by healthy controls [6]. The motif XPEFXGSXX was discovered and inferred to correspond to VPEFKGSLP in *Staphylococcus aureus* using protein database searches [45]. For *Poliovirus 1*, the epitope PALTAVETGATNPL was found to be a cross-reactive epitope in many enteroviruses [35]. The remainder of the literature epitopes were obtained directly from IEDB.

### Sequence processing

All software files are posted on GitHub (https://github.com/mlpaull/KTOPE) and all 278 antibody-binding peptide files are available from the Dryad Digital Repository (https://doi.org/10.5061/dryad.v7d0350). The imune-processor.jar file is available for research, non-profit, and non-commercial use, but requires a license for commercial use. All other software is available under the MIT license. The algorithms for generating nonredundant sequence lists from FASTQ files, outputting enrichment values for subsequences, and exhaustively calculating k-mer statistics were adapted from IMUNE (imune-processor.jar and calculate-patterns.jar) [29]. We added the capability to start with lists of peptides rather than NGS data. The enrichment of a k-mer is defined as the ratio of the number of observations of the k-mer to the “expected” number of observations. The “expected” value is calculated as the product of the total number of sequences, the number of frames the k-mer could fit in the sequences, and the probability of the k-mer appearing based on amino acid usage. If a k-mer’s enrichment is above the “enrichment minimum”, it is used in K-TOPE. The enrichment minimum was chosen as 2.0 for this study to reduce the dataset to only k-mers observed at least twice as often as expected. K-mers need to be calculated only once per specimen. All interaction with IMUNE-derived code is through a Python module which sets up a folder hierarchy and acts as a wrapper for IMUNE-derived code (imuneprocessor.py). These programs are memory and hard-drive intensive and it is recommended to have at least 16 GB of free RAM and 100 GB of hard-drive space. Analysis was carried out on a Dell Optiplex 9020 with an Intel^®^ Core^™^ i7-4790 CPU @ 3.60 GHz, 64-bit operating system, and 32.0 GB of RAM. Processing FASTQ files into subsequences from 12 specimens, each containing approximately 1.5 million unique sequences, required 2.3 hours and calculating k-mer enrichment required 7.7 minutes. The duration of these calculations scales approximately linearly with the number of specimens and sequences.

### K-TOPE algorithm

The K-TOPE algorithm (S1 Code) is written in Python 3.6 (KTOPE.py). A usage guide for KTOPE is available (S2 Text). First, there is a RAM-intensive step of loading k-mer enrichment data into memory as a dictionary. The enrichment dictionary for 250 specimens required approximately 4 GB of RAM. Then, a protein of interest is chosen for analysis and its sequence is loaded. This protein is tiled into k-mers of a set length. For this study, 5-mers were used. Each position in the protein sequence is assigned a frequency counter that starts at 0. The frequency counter of each sequence position contained in an enriched k-mer is incremented by the logarithm base 2 of the k-mer’s enrichment. For instance, if 3 k-mers that overlapped at a position had enrichments of 2, 4, and 8, the frequency for that position would be log_2_ 2 + log_2_ 4 + log_2_ 8 = 6. The frequency counters are compiled into a histogram which is smoothed using a moving window. For this analysis, to provide adequate smoothing, the window had width 7 and used linear weighting with 1 in the center and 0.1 at the edges. Minima and maxima are identified in the smoothed histogram. All intervals between 2 minima that contain a maximum are used to define epitopes. Epitopes were limited to a minimum length of 6 and a maximum length of 15 to roughly approximate the size of actual linear epitopes [22]. Epitopes are scored using the area under the curve of the un-smoothed histogram. To assign statistical significance to each epitope, the epitope’s score is ranked in a list of scores for epitopes of the same length generated through an analysis of 10,000 random proteins. This rank is reported as a percentile in the distribution of random protein epitope scores. For this study, an epitope percentile threshold of 95% was used. For 12 specimens, analysis of 10,000 random proteins required 10.0 minutes.

After determining epitopes for individual specimens, K-TOPE can determine consensus epitopes for a population. Each epitope is characterized by a “centroid” which is the weighted central position of the epitope, indexed as a position in the protein sequence. Centroids for all epitopes that meet the epitope percentile threshold are compiled. They are then clustered using k-means to associate close centroids with the KMeans function from scitkit-learn [69]. A representative epitope is made for each cluster and kept if it meets a minimum prevalence in the population. Closely overlapping epitopes are removed and the final list is sorted by prevalence. Consensus epitopes can be determined for each protein in a proteome, generating a list of epitopes prevalent in a population. Determination of consensus epitopes for the *Rhinovirus A* genome polyprotein (P07210) for 250 specimens required 24.4 seconds. The proteome searches for viruses with human tropism, *Staphylococcus*, and *Streptococcus* for 250 specimens required 3.1, 2.3, and 1.9 hours, respectively.

We calculated expected membership of epitope groups by multiplying the proportions of the population that bound each epitope. For example, if epitope 1 was bound by 32% of the population and epitope 2 was bound by 67%, then the expected membership of epitope group ‘1+2’ would be 21%. We ranked the overlaps between K-TOPE derived epitopes and literature epitopes in a list of 10,000 randomly generated epitope overlaps to determine a p-value. To remove redundant epitopes found in the proteome searches, we used the PAM30 similarity matrix to align two epitopes and compare each position to calculate a similarity score. Epitopes that had similarity scores >10, were in the same protein, and were from different organisms were considered redundant. We removed the less prevalent of the two redundant epitopes.

The HSV analysis used “disease” group specimens to predict epitopes and “control” group specimens to subtract epitopes. Epitopes were predicted in the disease group that met the epitope percentile threshold (95%) and the minimum prevalence (30%). Then, all disease epitopes were evaluated in the control group. For an epitope to be considered disease-specific, its score had to be below the epitope percentile threshold (80%) in all control specimens. To predict HSV2-specific epitopes that were also in the HSV1 proteome, we identified epitopes that exactly matched a subsequence in an HSV1 protein.

### Data visualization

Fig 1 was created using Inkscape. Histograms and heat maps were generated using the Matplotlib python module [70]. Bar graphs were generated using GraphPad Prism 7.

## Supporting information

S1 Fig A comparison of histograms generated by K-TOPE when antibodies were added to serum or buffer. Histograms were generated for antibodies against cMyc (P01106), V5 (P11207), and amyloid beta (P05067). The most prominent peaks were present regardless of whether antibodies were added to serum or buffer. This suggests that the binding signature of a single antibody was not obscured by the many other antibody specificities present in serum.
(TIF)

S2 Fig The number of epitopes generated as a function of varying the epitope percentile threshold. Epitopes were generated for 250 specimens using the *Rhinovirus A* genome polyprotein (P07210) with the prevalence fixed at 30%. The base 10 logarithm of the number of epitopes appeared to decrease linearly with increasing epitope percentile threshold. The value 95% was chosen for analysis because it corresponds to a p-value of 0.05 and ensures that the total number of epitopes predicted was of order one. By predicting a total number of epitopes of order one, fewer false positives should to be included in this analysis.
(TIF)

S3 Fig The number of epitopes generated as a function of varying the prevalence. Epitopes were generated for 250 specimens using the *Rhinovirus A* genome polyprotein (P07210) with the epitope percentile threshold fixed at 95%. The base 10 logarithm of the number of epitopes appeared to decrease exponentially with increasing prevalence. There were 123 epitopes bound by at least one member of the group. The value 30% was chosen arbitrarily from the prevalence values that predicted a total number of epitopes of order one. By predicting a total number of epitopes of order one, fewer false positives should to be included in this analysis.
(TIF)

S4 Fig Heat map of epitopes predicted for 43 *Enterovirus* strains. Analyzing multiple strains of *Enterovirus* revealed that the epitopes found for the *Rhinovirus A* strain analyzed in Fig 3 were found in multiple enteroviruses. The 4 epitopes in Fig 3 were similar to epitopes in other *Enterovirus* strains, as demonstrated by the bands at approximately positions 212–221, 569–578, 577–590, and 602–613 (respectively corresponding to epitopes 1, 2, 3, and 4). Epitopes 1, 2, and 4 were only found in *Rhinovirus*, whereas epitope 3 was found in many *Enterovirus* strains. The heat map was restricted to positions 0–700 to show relevant epitopes. A binary decision was made for each position in each protein to determine whether it was in an epitope.
(TIF)

S1 Table The expected and actual membership of different epitope groups. The expected membership of epitope groups was calculated by multiplying the proportions of the population that bound each epitope. For example, if epitope 1 was bound by 32% of the population and epitope 2 was bound by 67%, then the expected membership of epitope group ‘1+2’ would be 21%. Note that specimens in groups *only* bound the epitopes in the groups e.g. specimens in group ‘1’ did not bind ‘2’ or ‘3’. Most of the actual and expected membership values agreed except for the ‘1+2+3’, ‘3+4’, ‘1+2+4’, ‘1+2+3+4’, and the ‘None’ groups which had higher membership than expected and the ‘1+3’ group which had lower membership than expected. Additionally, the group targeting only epitope 4 was 40% smaller than expected suggesting that it was generally bound along with other epitopes. All groups that had percent differences equal to or greater than 50% are in bold.
(DOCX)

S2 Table The average age for each epitope group. The average age for the 138 specimens for which there was age data was 35. The ‘None’ group had an average age of 52 which was approximately 50% higher than the average age of 35 (in bold). Additionally, specimens targeting 3 or more epitopes had an average age of 17 (in bold), which was approximately 50% lower than the average age of 35. This discrepancy suggests that older people targeted fewer *Rhinovirus A* epitopes. The average age is given with the standard deviation.
(DOCX)

S3 Table Epitopes predicted for 43 *Enterovirus* strains. Analyzing multiple strains of *Enterovirus* revealed that the epitopes found for the *Rhinovirus A* strain analyzed in Fig 3 were found in multiple enteroviruses. Particularly, there were 31 strains with epitopes similar to epitope 3 in Fig 3 (APALDAAETGHT). Additionally, there were 3, 3, and 5 strains with epitopes similar to epitopes 1 (QNPVENYI), 2 (DSVLEVLVVPN), and 4 (NHTHPGEQG) from Fig 3, respectively. Epitopes 1, 2, and 4 were found in multiple rhinovirus strains suggesting that these epitopes were *Rhinovirus*-specific, but not *Enterovirus*-specific. Similarity comparisons used the PAM30 similarity matrix with similarity defined as a similarity score > 10.
(DOCX)

S4 Table Validating K-TOPE epitopes with prior studies. Twelve of the epitopes (in bold) were similar to epitopes found in prior studies with p-values of < 0.05. However, 30 of the epitopes were in proteins with no reported epitopes, and 3 epitopes were in organisms with no reported epitopes. Only 6 of the epitopes were in well-characterized proteins but were not found in the literature, suggesting that these epitopes were false positives or novel epitopes. Additionally, only two bacterial epitopes were in previously described proteins, suggesting that the remainder of the bacterial proteins were false positives or novel antigens. Epitopes 2, 3, and 4 differ slightly from those in Fig 4 because Fig 4 shows analysis for the most annotated accessions of these antigens, rather than the accessions used in K-TOPE analysis. Epitopes 4 and 11 were noted as part of the "GAGA" repeat region of EBNA1 and due to their frequency in the sequence, were not tested for significance. For epitopes 7, 17, and 31, the literature protein sequence did not match the protein sequence used for KTOPE searches. Instead, for these epitopes, the following accessions were used, respectively, P07210, A0A455KI32, and P0DF97 generating new epitopes DSVLNEVLVVPN, PALTAVETGHT, and KTDDMLNSND. Epitopes 1 and 7 matched the same literature epitope (NPVENYIDSVLNEVLVVPNIQ) and epitopes 2 and 17 matched similar literature epitopes (PALTAVETGATNPL and EAIPALTAVETGHTSQV). This suggests that the epitopes within each pair may be highly similar or identical. IEDB had 13 overlapping epitopes recorded for the protein streptolysin O, although for this analysis we chose the first of these epitopes (epitope 31). Surprisingly, epitope 20 in Murray Valley encephalitis had a corresponding literature epitope, but given the rarity of this virus, this was likely a coincidence. Each epitope was searched in IEDB by specifying the sequence with 70% BLAST similarity, the organism, the antigen name, positive assays only, and B Cell assays.
(DOCX)

S5 Table Eight HSV2-specific epitopes were also in the HSV1 proteome. (DOCX)

S6 Table Analysis of the HSV1 proteome predicted 30 epitopes that were bound by at least 10% of the 250 specimens. Notably, epitope 1 (FVLPHWYM) contains the 3-mer LPH which is also in the first epitope of the HSV1 specific epitopes, RIRLPHI (Table 5). Additionally, epitope 21 (PMPSLTA) contains the 4-mer PMPS which is also in epitope 2 of the HSV1-specific epitopes, PMPSIGLEE. A nearly exact match was found for epitope 18 (AAFVNDYS), which is highly similar to epitope 3 in the HSV1-specific epitope list, CAAFVNDYSLV. Thus, 3 of the 4 epitopes discovered from an HSV1-infected population had similarities to epitopes found using a general population. Additionally, 3 of the 4 antigens predicted from analyzing the 250 specimens were also predicted using the HSV1-infected specimens (major capsid protein, envelope glycoprotein G, and envelope glycoprotein D). Fewer epitopes were identified for the HSV1-infected specimens than the 250 specimens due to the group size disparity (10 specimens vs 250 specimens) and since the epitopes predicted from the HSV1-infected specimens used HSV2-infected specimens as controls. It is likely that the epitopes predicted for the HSV1 specimens and the 250 specimens did not match exactly because epitopes predicted from people with active HSV1 infections may not be identical to those predicted from people with latent HSV1 infections. Although the prevalence of HSV1 is nearly 50%, we did not find epitopes with a prevalence this high, likely because there are a variety of HSV1 epitopes that collectively indicate a prevalence of 50%. Hence, individual epitopes may only have prevalence values of 10–30%.
(DOCX)

S1 Code KTOPE software, written in Python 3.6. (TXT)

S1 Text Justification of conducting analysis with 5-mers. (DOCX)

S2 Text KTOPE usage guide. (DOCX)
