# Supplementary figures and images for: A general approach for predicting protein epitopes targeted by antibody repertoires using whole proteomes
Source: PLoS One. 2019 Sep 6;14(9):e0217668. doi: 10.1371/journal.pone.0217668 (PMC6730857; doi:10.1371/journal.pone.0217668)

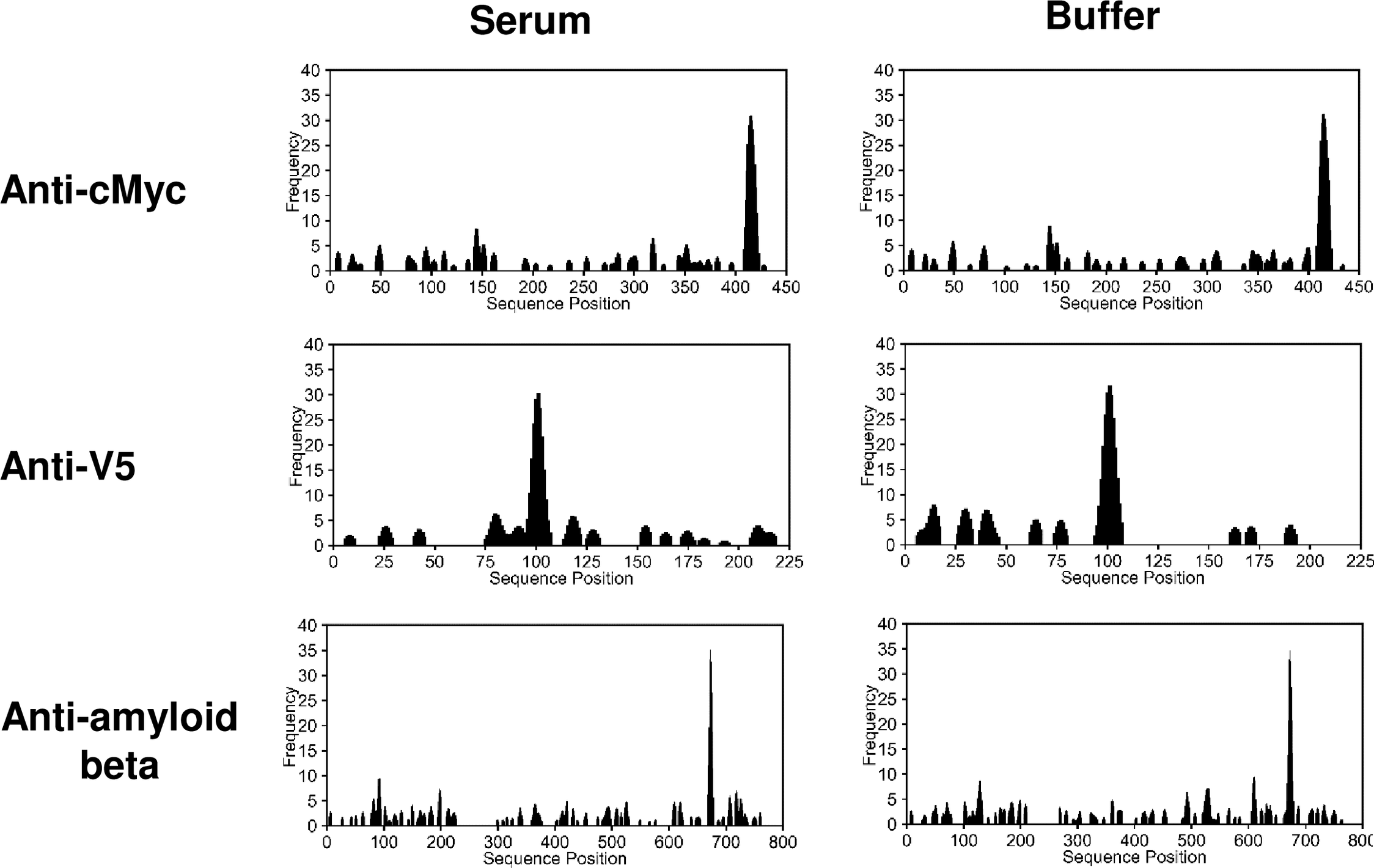

Supplement: S1 Fig — Histograms were generated for antibodies against cMyc (P01106), V5 (P11207), and amyloid beta (P05067). The most prominent peaks were present regardless of whether antibodies were added to serum or buffer. This suggests that the binding signature of a single antibody was not obscured by the many other antibody specificities present in serum. (TIF) [file pone.0217668.s001.tif]

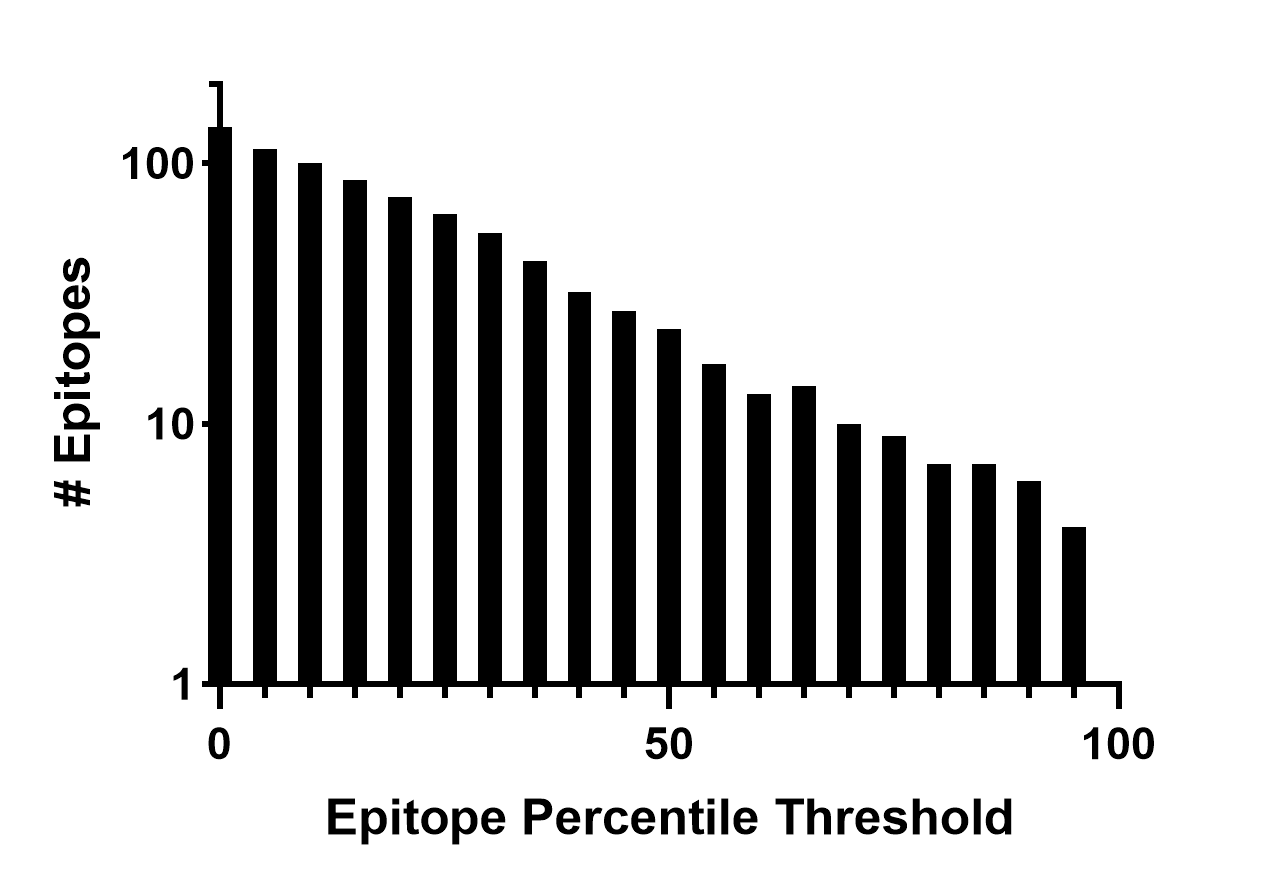

Supplement: S2 Fig — Epitopes were generated for 250 specimens using the Rhinovirus A genome polyprotein (P07210) with the prevalence fixed at 30%. The base 10 logarithm of the number of epitopes appeared to decrease linearly with increasing epitope percentile threshold. The value 95% was chosen for analysis because it corresponds to a p-value of 0.05 and ensures that the total number of epitopes predicted was of order one. By predicting a total number of epitopes of order one, fewer false positives should to be included in this analysis. (TIF) [file pone.0217668.s002.tif]

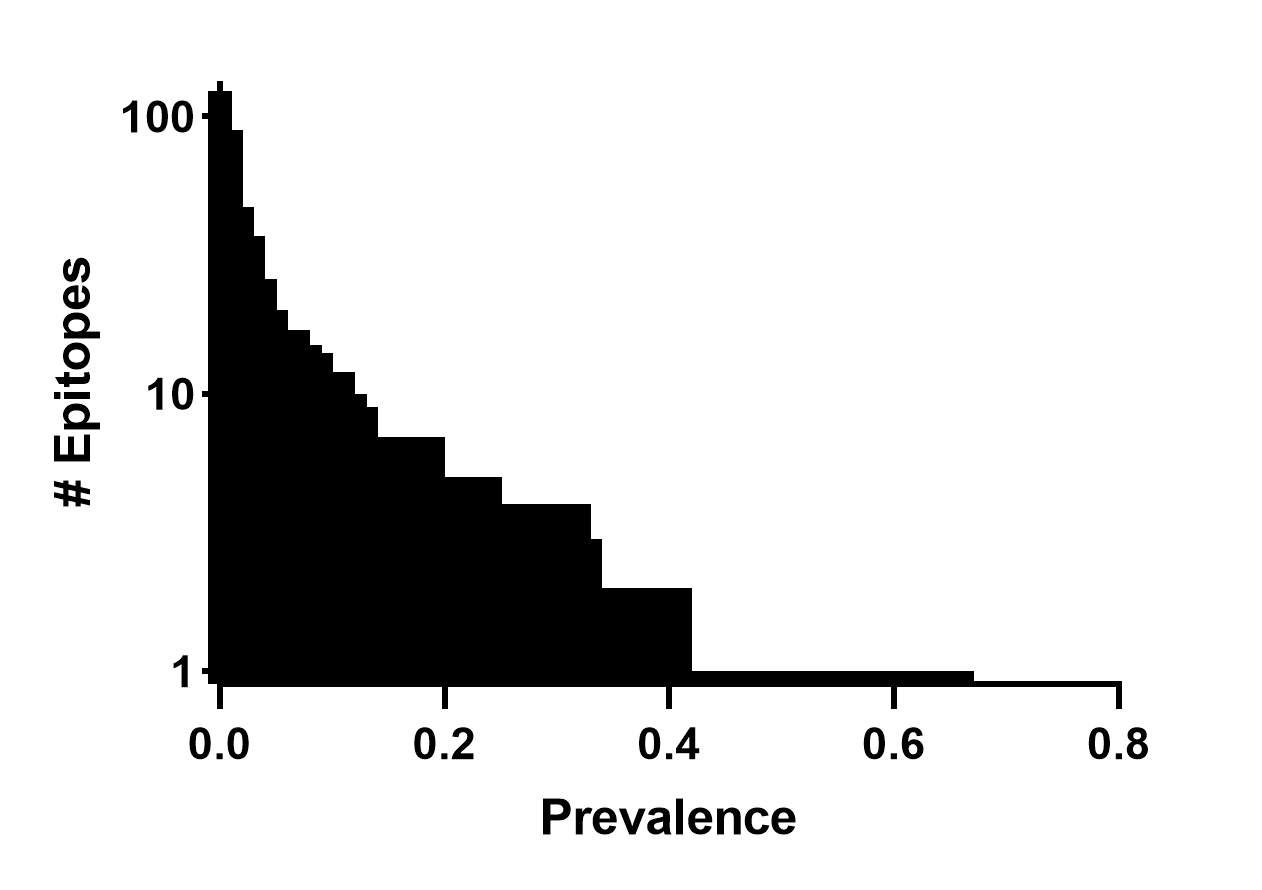

Supplement: S3 Fig — Epitopes were generated for 250 specimens using the Rhinovirus A genome polyprotein (P07210) with the epitope percentile threshold fixed at 95%. The base 10 logarithm of the number of epitopes appeared to decrease exponentially with increasing prevalence. There were 123 epitopes bound by at least one member of the group. The value 30% was chosen arbitrarily from the prevalence values that predicted a total number of epitopes of order one. By predicting a total number of epitopes of order one, fewer false positives should to be included in this analysis. (TIF) [file pone.0217668.s003.tif]

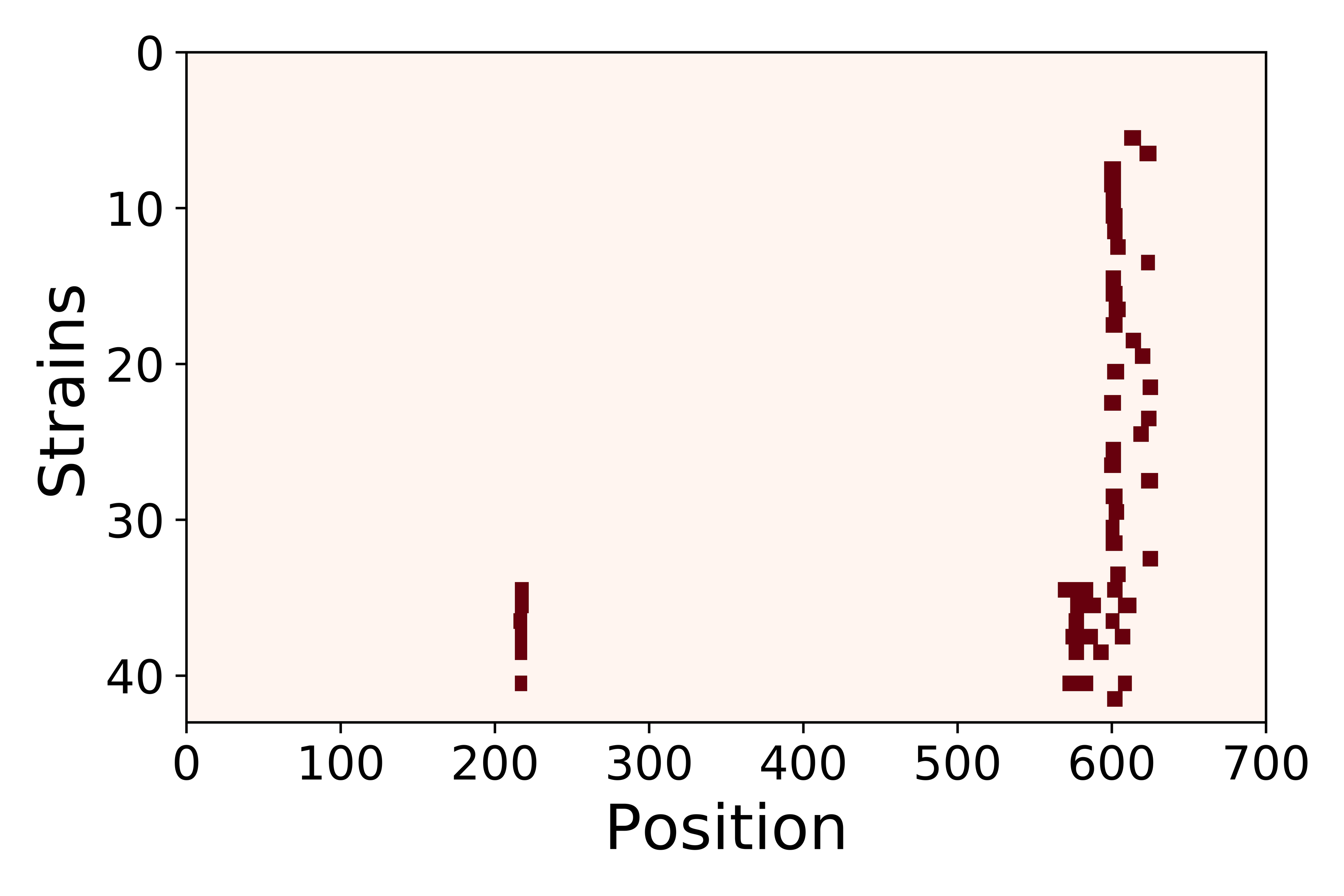

Supplement: S4 Fig — Analyzing multiple strains of Enterovirus revealed that the epitopes found for the Rhinovirus A strain analyzed in Fig 3 were found in multiple enteroviruses. The 4 epitopes in Fig 3 were similar to epitopes in other Enterovirus strains, as demonstrated by the bands at approximately positions 212–221, 569–578, 577–590, and 602–613 (respectively corresponding to epitopes 1, 2, 3, and 4). Epitopes 1, 2, and 4 were only found in Rhinovirus, whereas epitope 3 was found in many Enterovirus strains. The heat map was restricted to positions 0–700 to show relevant epitopes. A binary decision was made for each position in each protein to determine whether it was in an epitope. (TIF) [file pone.0217668.s004.tif]
