# Supplementary material for: A general approach for predicting protein epitopes targeted by antibody repertoires using whole proteomes
Source: PLoS One. 2019 Sep 6;14(9):e0217668. doi: 10.1371/journal.pone.0217668 (PMC6730857; doi:10.1371/journal.pone.0217668)
Supplement: S1 Table — The expected membership of epitope groups was calculated by multiplying the proportions of the population that bound each epitope. For example, if epitope 1 was bound by 32% of the population and epitope 2 was bound by 67%, then the expected membership of epitope group ‘1+2’ would be 21%. Note that specimens in groups only bound the epitopes in the groups e.g. specimens in group ‘1’ did not bind ‘2’ or ‘3’. Most of the actual and expected membership values agreed except for the ‘1+2+3’, ‘3+4’, ‘1+2+4’, ‘1+2+3+4’, and the ‘None’ groups which had higher membership than expected and the ‘1+3’ group which had lower membership than expected. Additionally, the group targeting only epitope 4 was 40% smaller than expected suggesting that it was generally bound along with other epitopes. All groups that had percent differences equal to or greater than 50% are in bold. (DOCX) [file pone.0217668.s005.docx]

# S1 Table

S1 Table. The expected and actual membership of different epitope groups.

The expected membership of epitope groups was calculated by multiplying the proportions of the population that bound each epitope. For example, if epitope 1 was bound by 32% of the population and epitope 2 was bound by 67%, then the expected membership of epitope group ‘1+2’ would be 21%. Note that specimens in groups *only* bound the epitopes in the groups e.g. specimens in group ‘1’ did not bind ‘2’ or ‘3’. Most of the actual and expected membership values agreed except for the ‘1+2+3’, ‘3+4’, ‘1+2+4’, ‘1+2+3+4’, and the ‘None’ groups which had higher membership than expected and the ‘1+3’ group which had lower membership than expected. Additionally, the group targeting only epitope 4 was 40% smaller than expected suggesting that it was generally bound along with other epitopes. All groups that had percent differences equal to or greater than 50% are in bold.

| Group | Actual | Expected | Percent Difference |
| --- | --- | --- | --- |
| 1 | 10 | 10 | 0% |
| 2 | 42 | 44 | -5% |
| 3 | 10 | 11 | -9% |
| 4 | 9 | 15 | -40% |
| 1+2 | 14 | 20 | -30% |
| 1+3 | **2** | **5** | **-60%** |
| 2+3 | 23 | 22 | 5% |
| 3+4 | **11** | **7** | **57%** |
| 1+4 | 6 | 7 | -14% |
| 2+4 | 30 | 30 | 0% |
| 1, 2, 3 | **26** | **10** | **160%** |
| 1, 2, 4 | **21** | **14** | **50%** |
| 1, 3, 4 | 2 | 3 | -33% |
| 2, 3, 4 | 11 | 15 | -27% |
| 1, 2, 3, 4 | **13** | **7** | **86%** |
| None | **33** | **21** | **57%** |
