# Supplementary material for: A general approach for predicting protein epitopes targeted by antibody repertoires using whole proteomes
Source: PLoS One. 2019 Sep 6;14(9):e0217668. doi: 10.1371/journal.pone.0217668 (PMC6730857; doi:10.1371/journal.pone.0217668)
Supplement: S2 Table — The average age for the 138 specimens for which there was age data was 35. The ‘None’ group had an average age of 52 which was approximately 50% higher than the average age of 35 (in bold). Additionally, specimens targeting 3 or more epitopes had an average age of 17 (in bold), which was approximately 50% lower than the average age of 35. This discrepancy suggests that older people targeted fewer Rhinovirus A epitopes. The average age is given with the standard deviation. (DOCX) [file pone.0217668.s006.docx]

# S2 Table

S2 Table. The average age for each epitope group.

The average age for the 138 specimens for which there was age data was 35. The ‘None’ group had an average age of 52 which was approximately 50% higher than the average age of 35 (in bold). Additionally, specimens targeting 3 or more epitopes had an average age of 17 (in bold), which was approximately 50% lower than the average age of 35. This discrepancy suggests that older people targeted fewer *Rhinovirus A* epitopes. The average age is given with the standard deviation.

| **Group** | **Average Age** | **Group Size** |
| --- | --- | --- |
| 1 | 49±19 | 3 |
| 2 | 20±23 | 22 |
| 3 | 38±20 | 4 |
| 4 | 40±20 | 3 |
| 1+2 | 33±27 | 5 |
| 1+3 | 21±0 | 1 |
| 1+4 | 10±2 | 3 |
| 2+3 | 34±27 | 12 |
| 2+4 | 30±27 | 13 |
| 3+4 | 37±24 | 7 |
| 1+2+3 | 20±14 | 7 |
| 1+2+4 | 15±11 | 9 |
| 1+3+4 | 57±0 | 1 |
| 2+3+4 | 14±9 | 8 |
| 1+2+3+4 | 12±5 | 4 |
| **3 or more epitopes** | **17±13** | **29** |
| **None** | **52±22** | **16** |
| **All Specimens** | **35±27** | **138** |
