# Supplementary material for: A general approach for predicting protein epitopes targeted by antibody repertoires using whole proteomes
Source: PLoS One. 2019 Sep 6;14(9):e0217668. doi: 10.1371/journal.pone.0217668 (PMC6730857; doi:10.1371/journal.pone.0217668)
Supplement: S3 Table — Analyzing multiple strains of Enterovirus revealed that the epitopes found for the Rhinovirus A strain analyzed in Fig 3 were found in multiple enteroviruses. Particularly, there were 31 strains with epitopes similar to epitope 3 in Fig 3 (APALDAAETGHT). Additionally, there were 3, 3, and 5 strains with epitopes similar to epitopes 1 (QNPVENYI), 2 (DSVLEVLVVPN), and 4 (NHTHPGEQG) from Fig 3, respectively. Epitopes 1, 2, and 4 were found in multiple rhinovirus strains suggesting that these epitopes were Rhinovirus-specific, but not Enterovirus-specific. Similarity comparisons used the PAM30 similarity matrix with similarity defined as a similarity score > 10. (DOCX) [file pone.0217668.s007.docx]

# S3 Table

S3 Table. Epitopes predicted for 43 *Enterovirus* strains. Analyzing multiple strains of *Enterovirus* revealed that the epitopes found for the *Rhinovirus A* strain analyzed in Fig 3 were found in multiple enteroviruses. Particularly, there were 31 strains with epitopes similar to epitope 3 in Fig 3 (APALDAAETGHT). Additionally, there were 3, 3, and 5 strains with epitopes similar to epitopes 1 (QNPVENYI), 2 (DSVLEVLVVPN), and 4 (NHTHPGEQG) from Fig 3, respectively. Epitopes 1, 2, and 4 were found in multiple rhinovirus strains suggesting that these epitopes were *Rhinovirus*-specific, but not *Enterovirus*-specific. Similarity comparisons used the PAM30 similarity matrix with similarity defined as a similarity score > 10.

| **Epitope** | **Organism** | **Accession** | **Prevalence** |
| --- | --- | --- | --- |
| DSVLNEVLVVPN | Human rhinovirus A serotype 89 (strain 41467-Gallo) (HRV-89) | P07210 | 0.668 |
| EVLNEVLVVPNIN | Human rhinovirus 2 OX=12130 | P04936 | 0.664 |
| EVLNEVLVVPNI | Human rhinovirus 16 (HRV-16) | Q82122 | 0.664 |
| VLNEVLVVPNIK | Human rhinovirus 1B OX=12129 | P12916 | 0.656 |
| PALTAAETG | Echovirus 12 (strain Travis) | Q66575 | 0.588 |
| VPALTAAETG | Echovirus 9 (strain Barty) OX=103914 | Q66577 | 0.58 |
| VPALTAAETG | Echovirus 9 (strain Hill) OX=103915 | Q66849 | 0.58 |
| VPALTAAETG | Echovirus 16 (strain Harrington) | Q66790 | 0.572 |
| PALTAAETGH | Coxsackievirus B3 (strain Woodruff) OX=103904 | Q66282 | 0.556 |
| PALTAAETGHT | Coxsackievirus B1 (strain Japan) OX=103902 | P08291 | 0.54 |
| PALTAAETGHT | Swine vesicular disease virus (strain UKG/27/72) OX=12077 | P13900 | 0.54 |
| PALTAAETGHT | Swine vesicular disease virus (strain H/3 '76) OX=12076 | P16604 | 0.54 |
| PALTAAETGHT | Coxsackievirus B5 (strain Peterborough / 1954/UK/85) OX=103907 | Q03053 | 0.54 |
| VPALTAVETG | Echovirus 11 (strain Gregory) OX=31705 | P29813 | 0.54 |
| IPALTAAETG | Coxsackievirus B3 (strain Nancy) OX=103903 | P03313 | 0.536 |
| IPALTAAETGH | Echovirus 6 (strain Charles) OX=103913 | Q66474 | 0.532 |
| VPALTAVETGH | Echovirus 5 (strain Noyce) OX=176283 | Q9YLJ1 | 0.524 |
| VPALTAVETGH | Coxsackievirus B6 (strain Schmitt) OX=231474 | Q9QL88 | 0.52 |
| VPALTAVETGH | Coxsackievirus A9 (strain Griggs) OX=12068 | P21404 | 0.516 |
| PALTAVETGHT | Coxsackievirus B4 (strain JVB / Benschoten / New York/51) OX=103906 | P08292 | 0.492 |
| PALTAVETGHT | Echovirus 30 (strain Bastianni) OX=176284 | Q9WN78 | 0.492 |
| KYTHPGEA | Human rhinovirus 16 (HRV-16) | Q82122 | 0.492 |
| IPALTAVETGH | Coxsackievirus B4 (strain E2) OX=103905 | Q86887 | 0.488 |
| SVPALTANE | Human rhinovirus 3 OX=44130 | Q82081 | 0.452 |
| YKYTHPGDR | Human rhinovirus 3 OX=44130 | Q82081 | 0.448 |
| NYTHPGETG | Human rhinovirus 2 OX=12130 | P04936 | 0.44 |
| EVPALTAVET | Coxsackievirus A21 (strain Coe) OX=12070 | P22055 | 0.432 |
| TFTHPGER | Human rhinovirus 14 OX=12131 | P03303 | 0.432 |
| NPVERYVDE | Human rhinovirus 16 (HRV-16) | Q82122 | 0.428 |
| EVPALTAVET | Coxsackievirus A24 (strain EH24/70) OX=36404 | P36290 | 0.424 |
| KEVPALTAVE | Poliovirus type 3 (strains P3/Leon/37 and P3/Leon 12A[1]B) OX=12088 | P03302 | 0.42 |
| NHTHPGEQG | Human rhinovirus A serotype 89 (strain 41467-Gallo) (HRV-89) | P07210 | 0.412 |
| NPVENYIDE | Human rhinovirus 1B OX=12129 | P12916 | 0.412 |
| EIPALTAVETG | Poliovirus type 1 (strain Mahoney) OX=12081 | P03300 | 0.392 |
| NLTHPGEA | Human rhinovirus 1B OX=12129 | P12916 | 0.392 |
| LTAVETGHTS | Echovirus 1 (strain Human/Egypt/Farouk/1951) OX=103908 | O91734 | 0.388 |
| IPALTAVETGA | Poliovirus type 1 (strain Sabin) OX=12082 | P03301 | 0.384 |
| APALDAAETG | Human rhinovirus 2 OX=12130 | P04936 | 0.376 |
| PVLTAVETGH | Coxsackievirus B2 (strain Ohio-1) | Q9YLG5 | 0.376 |
| ALTAVETGAT | Poliovirus type 2 (strain W-2) OX=12085 | P23069 | 0.372 |
| ALTAVETGAT | Poliovirus type 2 (strain Lansing) OX=12084 | P06210 | 0.372 |
| LEEVIVEKTK | Human rhinovirus 3 OX=44130 | Q82081 | 0.352 |
| LEEVIVEKTK | Human rhinovirus 14 OX=12131 | P03303 | 0.348 |
| AETGHTNKI | Human rhinovirus 16 (HRV-16) | Q82122 | 0.344 |
| APALDAAETGHT | Human rhinovirus A serotype 89 (strain 41467-Gallo) (HRV-89) | P07210 | 0.34 |
| PALQAAETGA | Coxsackievirus A16 (strain G-10) OX=69159 | Q65900 | 0.336 |
| SSGPKHTQKV | Human rhinovirus 14 OX=12131 | P03303 | 0.324 |
| QNPVENYI | Human rhinovirus A serotype 89 (strain 41467-Gallo) (HRV-89) | P07210 | 0.324 |
| LTAVETGAT | Poliovirus type 3 (strain 23127) OX=12087 | P06209 | 0.312 |
| VPALQAAETGA | Coxsackievirus A16 (strain Tainan/5079/98) OX=231417 | Q9QF31 | 0.304 |
| AQNPVENYID | Human rhinovirus 2 OX=12130 | P04936 | 0.304 |
| LLDAAETGHT | Human rhinovirus 1B OX=12129 | P12916 | 0.3 |
