# Supplementary material for: A general approach for predicting protein epitopes targeted by antibody repertoires using whole proteomes
Source: PLoS One. 2019 Sep 6;14(9):e0217668. doi: 10.1371/journal.pone.0217668 (PMC6730857; doi:10.1371/journal.pone.0217668)
Supplement: S4 Table — Twelve of the epitopes (in bold) were similar to epitopes found in prior studies with p-values of < 0.05. However, 30 of the epitopes were in proteins with no reported epitopes, and 3 epitopes were in organisms with no reported epitopes. Only 6 of the epitopes were in well-characterized proteins but were not found in the literature, suggesting that these epitopes were false positives or novel epitopes. Additionally, only two bacterial epitopes were in previously described proteins, suggesting that the remainder of the bacterial proteins were false positives or novel antigens. Epitopes 2, 3, and 4 differ slightly from those in Fig 4 because Fig 4 shows analysis for the most annotated accessions of these antigens, rather than the accessions used in K-TOPE analysis. Epitopes 4 and 11 were noted as part of the "GAGA" repeat region of EBNA1 and due to their frequency in the sequence, were not tested for significance. For epitopes 7, 17, and 31, the literature protein sequence did not match the protein sequence used for KTOPE searches. Instead, for these epitopes, the following accessions were used, respectively, P07210, A0A455KI32, and P0DF97 generating new epitopes DSVLNEVLVVPN, PALTAVETGHT, and KTDDMLNSND. Epitopes 1 and 7 matched the same literature epitope (NPVENYIDSVLNEVLVVPNIQ) and epitopes 2 and 17 matched similar literature epitopes (PALTAVETGATNPL and EAIPALTAVETGHTSQV). This suggests that the epitopes within each pair may be highly similar or identical. IEDB had 13 overlapping epitopes recorded for the protein streptolysin O, although for this analysis we chose the first of these epitopes (epitope 31). Surprisingly, epitope 20 in Murray Valley encephalitis had a corresponding literature epitope, but given the rarity of this virus, this was likely a coincidence. Each epitope was searched in IEDB by specifying the sequence with 70% BLAST similarity, the organism, the antigen name, positive assays only, and B Cell assays. (DOCX) [file pone.0217668.s008.docx]

**S4 Table**

**S4 Table. Validating K-TOPE epitopes with prior studies.** Twelve of the epitopes (in bold) were similar to epitopes found in prior studies with p-values of < 0.05. However, 30 of the epitopes were in proteins with no reported epitopes, and 3 epitopes were in organisms with no reported epitopes. Only 6 of the epitopes were in well-characterized proteins but were not found in the literature, suggesting that these epitopes were false positives or novel epitopes. Additionally, only two bacterial epitopes were in previously described proteins, suggesting that the remainder of the bacterial proteins were false positives or novel antigens. Epitopes 2, 3, and 4 differ slightly from those in Fig 4 because Fig 4 shows analysis for the most annotated accessions of these antigens, rather than the accessions used in K-TOPE analysis. Epitopes 4 and 11 were noted as part of the "GAGA" repeat region of EBNA1 and due to their frequency in the sequence, were not tested for significance. For epitopes 7, 17, and 31, the literature protein sequence did not match the protein sequence used for KTOPE searches. Instead, for these epitopes, the following accessions were used, respectively, P07210, A0A455KI32, and P0DF97 generating new epitopes DSVLNEVLVVPN, PALTAVETGHT, and KTDDMLNSND. Epitopes 1 and 7 matched the same literature epitope (NPVENYIDSVLNEVLVVPNIQ) and epitopes 2 and 17 matched similar literature epitopes (PALTAVETGATNPL and EAIPALTAVETGHTSQV). This suggests that the epitopes within each pair may be highly similar or identical. IEDB had 13 overlapping epitopes recorded for the protein streptolysin O, although for this analysis we chose the first of these epitopes (epitope 31). Surprisingly, epitope 20 in Murray Valley encephalitis had a corresponding literature epitope, but given the rarity of this virus, this was likely a coincidence. Each epitope was searched in IEDB by specifying the sequence with 70% BLAST similarity, the organism, the antigen name, positive assays only, and B Cell assays.

| # | KTOPE Epitope | Protein | Taxon | Accession | Prevalence | Validation | Literature Epitope | Source | P-Value |
| --- | --- | --- | --- | --- | --- | --- | --- | --- | --- |
| 1 | **DSVLNEVLVVPN** | **Genome polyprotein** | **Enterovirus** | **P07210** | **0.668** | **Found on IEDB** | **NPVENYIDSVLNEVLVVPNIQ** | **[1]** | **0.0018** |
| 2 | **PALTAAETG** | **Genome polyprotein** | **Enterovirus** | **Q66575** | **0.588** | **Found on IEDB**  **(Shown in Fig 4C)** | **PALTAVETGATNPL** | **[2]** | **0.003** |
| 3 | **GRRPFFHPV** | **Epstein-Barr nuclear antigen 1** | **Epstein-Barr virus (strain GD1)** | **Q1HVF7** | **0.524** | **Found on IEDB**  **(Shown in Fig 4A)** | **RRPFF** | **[3]** | **0.0031** |
| 4 | **AGAGGGAGA** | **Epstein-Barr nuclear antigen 1** | **Epstein-Barr virus (strain GD1)** | **Q1HVF7** | **0.516** | **Found on IEDB**  **(Shown in Fig 4A)** | **GA-repeat** | **[4]** | **N/A** |
| 5 | **KYTHPGEA** | **Genome polyprotein** | **Enterovirus** | **Q82122** | **0.492** | **Found on IEDB** | **KGNVNAGYKYTHPGE** | **[5]** | **0.0098** |
| 6 | VRRPFFSD | Protein UL84 | Human cytomegalovirus | P16727 | 0.452 | No literature match |  |  |  |
| 7 | **NPVERYVDE** | **Genome polyprotein** | **Enterovirus** | **Q82122** | **0.428** | **Found on IEDB** | **NPVENYIDSVLNEVLVVPNIQ** | **[1]** | **0.0017** |
| 8 | MVVPEFK | DNA-binding protein | Human mastadenovirus C | P03265 | 0.428 | No literature match |  |  |  |
| 9 | EVKLPHWTPT | Glycoprotein 42 | Epstein-Barr virus (strain GD1) | P03205 | 0.42 | None for protein |  |  |  |
| 10 | KPQPEKPK | Structural polyprotein | Mayaro virus | Q8QZ72 | 0.416 | None for organism |  |  |  |
| 11 | **GGAGAGGAGAGGG** | **Epstein-Barr nuclear antigen 1** | **Epstein-Barr virus (strain GD1)** | **P03211** | **0.412** | **Found on IEDB**  **(Shown in Fig 4A)** | **GA-repeat** | **[4]** | **N/A** |
| 12 | ININRPLE | Large structural protein | Lyssavirus | Q9QSP0 | 0.412 | None for protein |  |  |  |
| 13 | **RPSCIGCKG** | **Epstein-Barr nuclear antigen 1** | **Epstein-Barr virus (strain GD1)** | **P03211** | **0.404** | **Found on IEDB**  **(Shown in Fig 4A)** | **KRPSCIGCK** | **[6]** | **0.0056** |
| 14 | GAGAGAGGG | Packaging protein UL32 | Simplexvirus | P89455 | 0.376 | None for protein |  |  |  |
| 15 | DSVLNEVLVVPN | Genome polyprotein | Enterovirus | Q82081 | 0.352 | No literature match |  |  |  |
| 16 | KHTHPGI | Replication origin-binding protein | Human herpesvirus 3 | P09299 | 0.352 | None for protein |  |  |  |
| 17 | **AETGHTNKI** | **Genome polyprotein** | **Enterovirus** | **Q82122** | **0.344** | **Found on IEDB** | **EAIPALTAVETGHTSQV** | **[7]** | **0.0011** |
| 18 | YVFPHWITK | Envelope glycoprotein gp63 | Primate T-lymphotropic virus 3 | Q0R5Q9 | 0.34 | None for organism |  |  |  |
| 19 | KTTNTTTNT | Immediate-early protein 2 | Roseolovirus | Q9QJ16 | 0.34 | None for organism |  |  |  |
| 20 | **MAADKPTL** | **Genome polyprotein** | **Murray Valley encephalitis virus** | **P05769** | **0.34** | **Found on IEDB** | **AADKPTLDIRMMNIEA** | **[8]** | **0.0021** |
| 21 | SFIVPEFA | Virion membrane protein A16 | Orthopoxvirus | P16710 | 0.332 | None for protein |  |  |  |
| 22 | LVLPHWYMA | Cytoplasmic envelopment protein 1 | Simplexvirus | P89430 | 0.328 | None for protein |  |  |  |
| 23 | YVDDMLNDI | Large tegument protein deneddylase | Human herpesvirus 6A (strain Uganda-1102) | P52340 | 0.328 | None for protein |  |  |  |
| 24 | SSGPKHTQKV | Genome polyprotein | Enterovirus | P03303 | 0.324 | No literature match |  |  |  |
| 25 | PVPEFQA | Non-structural polyprotein | Semliki forest virus | P08411 | 0.316 | None for protein |  |  |  |
| 26 | VPVTPNIAI | Genome polyprotein | Hepatitis C virus | Q68749 | 0.304 | No literature match |  |  |  |
| 27 | LHRPALTA | Minor capsid protein L2 | Human papillomavirus type 34 | P36758 | 0.304 | No literature match |  |  |  |
| 28 | EHILNRPTG | RNA-directed RNA polymerase L | Crimean-Congo hemorrhagic fever orthonairovirus | Q6TQR6 | 0.304 | None for protein |  |  |  |
| 29 | GEFIGSE | Shutoff alkaline exonuclease | Human herpesvirus 8 | Q2HR95 | 0.3 | None for protein |  |  |  |
| 30 | LIPEFIGR | ATP-dependent Clp protease ATP-binding subunit ClpX | Streptococcus | P63793 | 0.512 | None for protein |  |  |  |
| 31 | **GQKMDDMLNS** | **Streptolysin O** | **Streptococcus** | **Q5XE40** | **0.436** | **Found on IEDB** | **AGQKTDDMLNSNDMI** | **[9]** | **0.0051** |
| 32 | QIPALDKPL | FMN-dependent NADH-azoreductase | Streptococcus | A4W2Z7 | 0.416 | None for protein |  |  |  |
| 33 | IADKPILD | UPF0154 protein SSU05_1707 | Streptococcus | A4VX34 | 0.392 | None for protein |  |  |  |
| 34 | TVADKPVA | Phenylalanine--tRNA ligase beta subunit | Streptococcus | Q5XCX3 | 0.36 | None for protein |  |  |  |
| 35 | RTPDKPT | Agglutinin receptor | Streptococcus | P16952 | 0.324 | None for protein |  |  |  |
| 36 | VVPNIWR | Putative 2-dehydropantoate 2-reductase | Streptococcus | P65666 | 0.32 | None for protein |  |  |  |
| 37 | LLNRPIHD | CCA-adding enzyme | Streptococcus | Q5M153 | 0.32 | None for protein |  |  |  |
| 38 | TLADKPEF | Autolysin | Streptococcus | P06653 | 0.308 | None for protein |  |  |  |
| 39 | **PTHYVPEFKGS** | **Extracellular matrix protein-binding protein emp** | **Staphylococcus** | **Q2FIK4** | **0.572** | **Found on IEDB**  **(Shown in Fig 4B)** | **VPEFKGSLP** | **[10]** | **0.019** |
| 40 | LIPEFIG | ATP-dependent Clp protease ATP-binding subunit ClpX | Staphylococcus | B9DNC0 | 0.508 | None for protein |  |  |  |
| 41 | NKPEFSGAT | 3-isopropylmalate dehydratase small subunit | Staphylococcus | Q4L7U3 | 0.436 | None for protein |  |  |  |
| 42 | NKNNKNNKN | Translation initiation factor IF-2 | Staphylococcus | Q4L5X1 | 0.372 | None for protein |  |  |  |
| 43 | KLGNIVPEYK | Extracellular matrix protein-binding protein emp | Staphylococcus | P0C6P1 | 0.36 | None for protein |  |  |  |
| 44 | KLCRICFRE | 30S ribosomal protein S14 type Z | Staphylococcus | Q5HM12 | 0.352 | None for protein |  |  |  |
| 45 | DFLNRPVD | Proline--tRNA ligase | Staphylococcus | Q4L5W5 | 0.348 | None for protein |  |  |  |
| 46 | EKNNNNNNNNS | Alkaline shock protein 23 | Staphylococcus | Q4L860 | 0.32 | None for protein |  |  |  |
| 47 | GVVPNISR | UvrABC system protein A | Staphylococcus | Q5HHQ9 | 0.312 | None for protein |  |  |  |
| 48 | LIPEFNQV | Homoserine kinase | Staphylococcus | Q8CSQ2 | 0.308 | None for protein |  |  |  |
| 49 | SPEFLGSQ | Undecaprenyl-diphosphatase | Staphylococcus | B9DK59 | 0.308 | None for protein |  |  |  |
| 50 | VGINRPTY | Putative glycosyltransferase TagX | Staphylococcus | O05154 | 0.308 | None for protein |  |  |  |
| 51 | VIPEFNND | Peptide chain release factor 2 | Staphylococcus | Q4L4H9 | 0.3 | None for protein |  |  |  |

**References**

1. Niespodziana K, Napora K, Cabauatan C, Focke-Tejkl M, Keller W, Niederberger V, et al. Misdirected antibody responses against an N-terminal epitope on human rhinovirus VP1 as explanation for recurrent RV infections. FASEB J. 2012;26: 1001–1008. doi:10.1096/fj.11-193557

2. Samuelson A, Forsgren M, Johansson BO, Wahren B. Molecular basis for serological cross-reactivity between enteroviruses. Clin Diagn Lab Immunol. 1994;1: 336–341.

3. Larman HB, Laserson U, Querol L, Verhaeghen K, Solimini NL, Xu GJ, et al. PhIP-Seq characterization of autoantibodies from patients with multiple sclerosis, type 1 diabetes and rheumatoid arthritis. J Autoimmun. 2013;43: 1–9. doi:10.1016/j.jaut.2013.01.013

4. Hecker M, Fitzner B, Wendt M, Lorenz P, Flechtner K, Steinbeck F, et al. High-density peptide microarray analysis of IgG autoantibody reactivities in serum and cerebrospinal fluid of multiple sclerosis patients. Mol Cell Proteomics. 2016;15: 1360–80. doi:10.1074/mcp.M115.051664

5. Sam Narean J, Glanville N, Nunn CM, Niespodziana K, Valenta R, Johnston SL, et al. Epitope mapping of antibodies induced with a conserved rhinovirus protein generating protective anti-rhinovirus immunity. Vaccine. Elsevier; 2019;37: 2805–2813. doi:10.1016/j.vaccine.2019.04.018

6. Elliott SE, Parchim NF, Kellems RE, Xia Y, Soffici AR, Daugherty PS. A pre-eclampsia-associated Epstein-Barr virus antibody cross-reacts with placental GPR50. Clin Immunol. 2016;168: 64–71. doi:10.1016/j.clim.2016.05.002

7. Härkönen T, Lankinen H, Davydova B, Hovi T, Roivainen M. Enterovirus infection can induce immune responses that cross-react with β-cell autoantigen tyrosine phosphatase IA-2/IAR. J Med Virol. 2002;66: 340–350. doi:10.1002/jmv.2151

8. Roehrig JT, Hunt AR, Johnson AJ, Hawkes RA. Synthetic peptides derived from the deduced amino acid sequence of the E-glycoprotein of Murray Valley encephalitis virus elicit antiviral antibody. Virology. 1989; doi:10.1016/0042-6822(89)90509-6

9. Mortensen R, Nissen TN, Fredslund S, Rosenkrands I, Christensen JP, Andersen P, et al. Identifying protective Streptococcus pyogenes vaccine antigens recognized by both B and T cells in human adults and children. Sci Rep. Nature Publishing Group; 2016;6: 22030. doi:10.1038/srep22030

10. Weber LK, Palermo A, Kügler J, Armant O, Isse A, Rentschler S, et al. Single amino acid fingerprinting of the human antibody repertoire with high density peptide arrays. J Immunol Methods. Elsevier B.V; 2017;443: 45–54. doi:10.1016/j.jim.2017.01.012
