# Supplementary material for: A general approach for predicting protein epitopes targeted by antibody repertoires using whole proteomes
Source: PLoS One. 2019 Sep 6;14(9):e0217668. doi: 10.1371/journal.pone.0217668 (PMC6730857; doi:10.1371/journal.pone.0217668)
Supplement: S5 Table — (DOCX) [file pone.0217668.s009.docx]

# S5 Table

Table S5. Eight HSV2-specific epitopes were also in the HSV1 proteome.

| **Epitope** | **Protein** | **Accession** | **Prevalence** |
| --- | --- | --- | --- |
| PLYARTTPAKF | Tegument protein UL47 | P89467 | 1 |
| TPLRYACVL | Tegument protein UL47 | P89467 | 1 |
| ANSPWAPVL | mRNA export factor | P28276 | 1 |
| QRLTPH | Large tegument protein deneddylase | P89459 | 1 |
| LRTNNL | Ribonucleoside-diphosphate reductase small subunit | P69521 | 0.917 |
| PSTPAMLNLG | Ribonucleoside-diphosphate reductase large subunit | P89462 | 0.667 |
| YFEEYAYS | Envelope glycoprotein B | P08666 | 0.417 |
| LDDFDL | Tegument protein VP16 | P68336 | 0.417 |
