# Supplementary material for: A general approach for predicting protein epitopes targeted by antibody repertoires using whole proteomes
Source: PLoS One. 2019 Sep 6;14(9):e0217668. doi: 10.1371/journal.pone.0217668 (PMC6730857; doi:10.1371/journal.pone.0217668)
Supplement: S6 Table — Notably, epitope 1 (FVLPHWYM) contains the 3-mer LPH which is also in the first epitope of the HSV1 specific epitopes, RIRLPHI (Table 5). Additionally, epitope 21 (PMPSLTA) contains the 4-mer PMPS which is also in epitope 2 of the HSV1-specific epitopes, PMPSIGLEE. A nearly exact match was found for epitope 18 (AAFVNDYS), which is highly similar to epitope 3 in the HSV1-specific epitope list, CAAFVNDYSLV. Thus, 3 of the 4 epitopes discovered from an HSV1-infected population had similarities to epitopes found using a general population. Additionally, 3 of the 4 antigens predicted from analyzing the 250 specimens were also predicted using the HSV1-infected specimens (major capsid protein, envelope glycoprotein G, and envelope glycoprotein D). Fewer epitopes were identified for the HSV1-infected specimens than the 250 specimens due to the group size disparity (10 specimens vs 250 specimens) and since the epitopes predicted from the HSV1-infected specimens used HSV2-infected specimens as controls. It is likely that the epitopes predicted for the HSV1 specimens and the 250 specimens did not match exactly because epitopes predicted from people with active HSV1 infections may not be identical to those predicted from people with latent HSV1 infections. Although the prevalence of HSV1 is nearly 50%, we did not find epitopes with a prevalence this high, likely because there are a variety of HSV1 epitopes that collectively indicate a prevalence of 50%. Hence, individual epitopes may only have prevalence values of 10–30%. (DOCX) [file pone.0217668.s010.docx]

# S6 Table

S6 Table. Analysis of the HSV1 proteome predicted 30 epitopes that were bound by at least 10% of the 250 specimens. Notably, epitope 1 (FVLPHWYM) contains the 3-mer LPH which is also in the first epitope of the HSV1 specific epitopes, RIRLPHI (Table 5). Additionally, epitope 21 (PMPSLTA) contains the 4-mer PMPS which is also in epitope 2 of the HSV1-specific epitopes, PMPSIGLEE. A nearly exact match was found for epitope 18 (AAFVNDYS), which is highly similar to epitope 3 in the HSV1-specific epitope list, CAAFVNDYSLV. Thus, 3 of the 4 epitopes discovered from an HSV1-infected population had similarities to epitopes found using a general population. Additionally, 3 of the 4 antigens predicted from analyzing the 250 specimens were also predicted using the HSV1-infected specimens (major capsid protein, envelope glycoprotein G, and envelope glycoprotein D). Fewer epitopes were identified for the HSV1-infected specimens than the 250 specimens due to the group size disparity (10 specimens vs 250 specimens) and since the epitopes predicted from the HSV1-infected specimens used HSV2-infected specimens as controls. It is likely that the epitopes predicted for the HSV1 specimens and the 250 specimens did not match exactly because epitopes predicted from people with active HSV1 infections may not be identical to those predicted from people with latent HSV1 infections. Although the prevalence of HSV1 is nearly 50%, we did not find epitopes with a prevalence this high, likely because there are a variety of HSV1 epitopes that collectively indicate a prevalence of 50%. Hence, individual epitopes may only have prevalence values of 10-30%.

| **#** | **Epitope** | **Protein** | **Accession** | **Prevalence** |
| --- | --- | --- | --- | --- |
| 1 | FVLPHWYM | Cytoplasmic envelopment protein 1 | P10191 | 0.32 |
| 2 | LSPALTALT | Ribonucleoside-diphosphate reductase small subunit | P10224 | 0.236 |
| 3 | VEVMVVPA | Envelope glycoprotein H | P06477 | 0.204 |
| 4 | LPFFNRPL | Major DNA-binding protein | P04296 | 0.204 |
| 5 | LRRRRRR | Envelope protein US9 | P06481 | 0.196 |
| 6 | ILNKPVFI | Tripartite terminase subunit 3 | P04295 | 0.192 |
| 7 | NTNTTTNS | E3 ubiquitin-protein ligase ICP0 | P08393 | 0.168 |
| 8 | TAMHTSKTD | Tegument protein UL55 | P10239 | 0.144 |
| 9 | PETPKPSHT | Serine/threonine-protein kinase US3 | P04413 | 0.14 |
| 10 | YPRNPVEFV | Envelope glycoprotein C | P10228 | 0.128 |
| 11 | PQPQPQPQ | Large tegument protein deneddylase | P10220 | 0.128 |
| 12 | SIQDAATPYHP | Envelope glycoprotein D | Q69091 | 0.124 |
| 13 | SRKPPNPTP | Envelope glycoprotein B | P10211 | 0.12 |
| 14 | VTQNKTTP | Envelope glycoprotein C | P10228 | 0.12 |
| 15 | PLVNRPA | E3 ubiquitin-protein ligase ICP0 | P08393 | 0.12 |
| 16 | INYQHTHKA | Serine/threonine-protein kinase UL13 | P04290 | 0.116 |
| 17 | EDILNDVL | Capsid vertex component 1 | P10201 | 0.116 |
| 18 | AAFVNDYS | Major capsid protein | P06491 | 0.112 |
| 19 | QPQPQPQ | Large tegument protein deneddylase | P10220 | 0.112 |
| 20 | RHTQKA | Envelope glycoprotein D | Q69091 | 0.112 |
| 21 | PMPSLTA | Envelope glycoprotein I | P06487 | 0.112 |
| 22 | DVEKDKPNR | Envelope glycoprotein G | P06484 | 0.108 |
| 23 | RLTALELI | Capsid vertex component 2 | P10209 | 0.108 |
| 24 | EPWPGAG | Major viral transcription factor ICP4 | P08392 | 0.108 |
| 25 | VIDKINA | Envelope glycoprotein B | P10211 | 0.104 |
| 26 | QLLHNTQA | Major capsid protein | P06491 | 0.1 |
| 27 | LREVLAARE | Large tegument protein deneddylase | P10220 | 0.1 |
| 28 | GPSHTAAPA | Major viral transcription factor ICP4 | P08392 | 0.1 |
| 29 | VCGRCCSII | Envelope glycoprotein K | P68331 | 0.1 |
| 30 | YKDTHHTP | Tegument protein UL55 | P10239 | 0.1 |
