# Supplementary material for: A general approach for predicting protein epitopes targeted by antibody repertoires using whole proteomes
Source: PLoS One. 2019 Sep 6;14(9):e0217668. doi: 10.1371/journal.pone.0217668 (PMC6730857; doi:10.1371/journal.pone.0217668)
Supplement: S1 Text — (DOCX) [file pone.0217668.s012.docx]

### **S1 Text: Justification of conducting analysis with 5-mers**

We chose to use 5-mers for K-TOPE analysis because they were the optimal k-mer length based on library coverage and enrichment dynamic range. Here, library coverage of a k-mer is defined as the percentage of all 20^k^ k-mers that are observed at least once in the 12-mer peptide library before selection. The library coverage percentages of all 12 k-mers is presented in S1A Text.

S1A Text: Library coverage for k-mers of varying length.

Note that the exact coverage for 5-mers is 99.99990625 %, thus only three out of 3.2 million 5-mers were not observed.

| K-mer Length | Percentage (%) |
| --- | --- |
| 1 | 100 |
| 2 | 100 |
| 3 | 100 |
| 4 | 100 |
| 5 | 100.0 |
| 6 | 96.0 |
| 7 | 25.0 |
| 8 | 1.3 |
| 9 | 5.3E-02 |
| 10 | 2.0E-03 |
| 11 | 6.6E-05 |
| 12 | 1.6E-06 |

As shown in S1A Text, 5-mers were the longest k-mers that still had virtually complete library coverage. While 6-mers could still be useful for analysis, they only had approximately 96% coverage. To increase coverage of the 6-mers, we would need to construct a larger library, conduct experiments with a larger oversampling of the library, or achieve greater NGS sequencing depth. To further illustrate the limitations of using 6-mers, we examined the 5-mer and 6-mer coverage after selection for a single specimen. In this set, there was 87.7% coverage of 5-mers and 15.1% coverage of 6-mers. The maximum possible 6-mer coverage for this specimen was 20.3%. Therefore, since K-TOPE analyzes a single specimen at a time, 6-mers will not have greater than approximately 20% coverage. In summary, to ensure that we avoided sparse datasets we conducted analysis with 5-mers.

Additionally, the length of a k-mer affects expected observations and therefore affects the dynamic range of enrichments. To illustrate, a k-mer with 1 observation and an expected value of 1 would have an enrichment of 1. In contrast, a k-mer with 1 observation and an expected value of 0.1 would have an enrichment of 10. In the case of low expected values, a single observation will lead to a high enrichment. However, a single observation could possibly be due to noise. Ideally, the expected value should be of order one such that a single observation will not lead to a high enrichment. To identify which k-mer length had an expected value of order one, we calculated the expected values for a set of 1.5 million 12-mer peptides with equal amino acid frequencies of 0.05 (S1B Text). The 5-mers were the only k-mer length with an expected value of order one, with an expected value of around 4. Note that the actual expected value varies with the amino acid frequencies and the number of sequences. Thus, for 6-mers to have an expected value of order one, we would require approximately 10 times more sequences per specimen. Thus, with the current number of sequences per specimen, k-mers of length 5 were optimal for K-TOPE.

S1B Text: The expected number of sequences for different k-mer lengths.

These calculations assumed a total of 1.5 million sequences and equal amino acid frequencies of 0.05.

Only 5-mers have an expected value of order 1.

| K-mer Length | Expected |
| --- | --- |
| 1 | 9.0E+05 |
| 2 | 4.1E+04 |
| 3 | 1.9E+03 |
| 4 | 84 |
| 5 | 3.8 |
| 6 | 0.16 |
| 7 | 7.0E-03 |
| 8 | 2.9E-04 |
| 9 | 1.2E-05 |
| 10 | 4.4E-07 |
| 11 | 1.5E-08 |
| 12 | 3.7E-10 |
