# Supplementary material for: A general approach for predicting protein epitopes targeted by antibody repertoires using whole proteomes
Source: PLoS One. 2019 Sep 6;14(9):e0217668. doi: 10.1371/journal.pone.0217668 (PMC6730857; doi:10.1371/journal.pone.0217668)
Supplement: S2 Text — (DOCX) [file pone.0217668.s013.docx]

**S2 Text: KTOPE Usage Guide**

Contents

[Installing programming packages 2](#_Toc8764237)

[Processing raw data using IMUNE processor 2](#_Toc8764238)

[Setting up processing run 2](#_Toc8764239)

[Processing an NGS file 4](#_Toc8764240)

[Processing peptide files 4](#_Toc8764241)

[Running command files 5](#_Toc8764242)

[Kmer calculation 6](#_Toc8764243)

[Kmer Tiling 7](#_Toc8764244)

[Making enrichment dictionaries 7](#_Toc8764245)

[Generating RandomStats 8](#_Toc8764246)

[Determining epitopes 9](#_Toc8764247)

[Determining consensus epitopes 11](#_Toc8764248)

[Determining epitopes for a list of proteins 12](#_Toc8764249)

[Identifying group-specific epitopes 14](#_Toc8764250)

# Installing programming packages

The IMUNE processor requires a 64-bit version of java. Java can be installed here: <https://java.com/en/download/manual.jsp>

The KTOPE code requires a Python 3.X 64-bit version. I recommend the Anaconda package: <https://www.continuum.io/downloads>

If 32-bit versions are installed, then there will be errors, as the RAM limitations of 32-bit versions will be quickly exceeded.

The following python packages will likely need to be installed using easy_install or pip install:

Biopython: <http://biopython.org/wiki/Download>

sklearn: <http://scikit-learn.org/stable/install.html>

The following modules are needed, but most are included in python installation packages:

os, time, subprocess, numpy, math, scipy, itertools, re, pickle, Bio, sklearn

Import KTOPE.py and imuneprocessor.py prior to starting.

# Processing raw data using IMUNE processor

## Setting up processing run

Raw data can be de-multiplexed FASTQ files generated by Illumina with the standard format e.g.:

@NS500585:86:H72CMBGXY:1:11101:17458:1078 1:N:0:CGTACTAG+NTCCTTAC

TTTGGCCAGTCTGGCCAGGGTGGATTCTCTCTGTCTGATTACATCTTCTACCATAAACAGGGAGGGCAGTCTGGG

+

AAAAAEEEEEEEEEEEEEEEEEEEAEEEEAEEEEEEEEEEEEEEEAEEEEEEEEEEEEEEEEEEEEAEEEEEEEE

In this case, there can be multiple FASTQ files for 1 sample, but there can not be sequences for multiple samples in 1 FASTQ file. This input is ideal for display systems such as bacterial or bacteriophage peptide display.

The second format for data is a list of peptide files, where the first line of the file has the sample specifications in the form PROJECT CLASSIFICATION NAME:

Peptide file example:

Project Classification Name

SIGYEHGSKGSNSKM

VQERGSKGSNNKVDM

SQPLYGQSGDGQSGQ

GCSTLVVPNLSVLSR

The imuneprocessor.py file is a python wrapper for the java code immune-processor.jar. The first step is to make a directory in which calculations and kmer data will be stored. This will be referred to as the “root directory”. To create a folder hierarchy, run the following commands:

#Put r in front of strings to avoid read as escape sequences

rootdirectory = r'C:\KTOPE'

imuneprocessor = IMUNE_processor(rootdirectory)

imuneprocessor.create_folders()

This will create a folder structure of the following folders:

*Commands*: Contains commands files which are read by immune-processor.jar

*logs*: calculate-kmers.jar calculation summaries are put into this folder

*Samples*: Subsequences are evaluated from sequences and put into a hierarchy of project\classification\sample\subsequences

*Results*: Calculation summaries are put in here for processing runs under “Processing” and evaluated kmers are put here for calculate-patterns.jar

*Proteins*: Pickled protein objects are kept here for proteins.

*Data*: General folder for raw data and analysis

After running these commands, copy the imune-processor.jar file and the calculate-patterns.jar file into the rootdirectory.

Note: All projects, classifications, and names for samples should have NO spaces or characters that can’t be used in file names. Also, there should be no spaces used in fastq file addresses. Strings are split on spaces, so any spaces in names or addresses will cause issues.

## Processing an NGS file

For processing data using NGS files, first make a datalabel file where each line has the format PROJECT CLASSIFICATION NAME FASTQLOCATION e.g.

Project Classification Name C:\KTOPE\Data\fastq1.fastq

Project Classification Name C:\KTOPE\Data\fastq2.fastq

With the address of the datalabel file, the following commands are run (change relevant parameters):

rootdirectory = r'C:\KTOPE'

commandname = r'Example_NGS_process’

#Annealing sequences before and after desired sequence

annealdict = {'before': 'GGCCAGTCTGGCCAGGGTGGA','after':'GGAGGGCAGTCTGGGCAG'}

datalabel = r'C:\KTOPE\Data\Example_datalabel.txt'

minseqlen = 15

maxseqlen = 15

imuneprocessor = IMUNE_processor(rootdirectory)

imuneprocessor.processor_NGS_cmd_gen(commandname,annealdict, datalabel,minseqlen,maxseqlen)

## Processing peptide files

The peptide files, in the format specified earlier, must be combined into a single file for use with imune_processor. Compile the addresses of all peptide files into a list called filelist. After being combined, a command file is made. The following code accomplishes these tasks:

rootdirectory = r'C:\KTOPE'

commandname = r'Example_peptide_process’

combinedfile = r'C:\KTOPE\Data\Peptide_Combined.txt'

minseqlen = 15

maxseqlen = 15

imuneprocessor = IMUNE_processor(rootdirectory)

imuneprocessor.processor_peptide_file_combine(filelist,combinedfile)

imuneprocessor.processor_peptide_cmd_gen(combinedfile,commandname,minseqlen,maxseqlen)

## Running command files

The command files generated using either method can be run on the command line:

java -Xmx12g -jar immune-processor.jar C:\KTOPE\Commands\Example_NGS_process.txt maxthreads 4

Or using the imuneprocessor.py code:

#Amount of RAM used in GB

memory = 12

#Number of threads used for calculations

maxthreads = 4

imuneprocessor.processing_cmdline_run(memory,maxthreads)

Note: Once the process is started in python, it can’t be terminated through python. The java process has to be terminated in the task manager and the python kernel must be quit. If a Results and/or Samples folder for this specific run has already been created, it must be deleted.

The default number of threads used is the number of logical cores divided by 2. The default RAM used is ¼ of the physical memory.

At the end of running the imune-processor.jar file, there should be subsequences in the sample folder corresponding to the input raw data.

The processing_cmdline_run function may not work if Java and Python are not in the PATH.

# Kmer calculation

Samples only need to be processed a single time into kmers. After that kmers can be determined from the sample subsequences multiple times for different parameter choices. Kmer calculation creates a dense semicolon delimited file which can then be used with KTOPE.py. The following parameters have to be specified:

*mindef*: minimum number of defined positions in kmer

*maxdef*: maximum number of defined positions in kmer

*minlen*: minimum length of kmers

*maxlen*: maximum length of kmers

*enrichmin*: Minimum enrichment cutoff for a kmer to be significant

The calculate-patterns.jar will calculate all kmers that meet these thresholds for instance kmers are created with the following parameters:

mindef,maxdef,minlen,maxlen = 4,5,4,5

kmers: NNNN, NNNNN, NXNNN, NNXNN, NNNXN

where N is one of the 20 amino acids, and X is a wildcard position.

For this paper 5 was used for all four parameters.

The imuneprocessor object has a samplelist attribute which is set when processing samples. When starting with calculating kmers, it has to be set with the initialize_samplelist which takes a samplelist file of the following format:

Project Classification Name1

Project Classification Name2

The following code is run to calculate kmers:

rootdirectory = r'C:\KTOPE

samplefolder = r’C:\KTOPE\Samples’

outputfolder = r'Example_kmers’

memory = 12

mindef,maxdef,minlen,maxlen = 5,5,5,5

enrichmin = 2.0

imuneprocessor = IMUNE_processor(rootdirectory)

imuneprocessor.initialize_samplelist(r"C:\KTOPE\Data\example_samplelist.txt”)

imuneprocessor.calculate_kmers_cmd_gen(samplefolder,outputfolder,mindef,maxdef,minlen,maxlen,enrichmin)

The command file created can then be run using python:

imuneprocessor.calculate_kmers_cmdline_run(memory)

Or on the command line:

java -Xmx12g -jar calculate-patterns.jar C:\KTOPE\Commands\Example_Kmers.txt

Note: Once the process is started in python, it can’t be terminated through python. The java process has to be terminated in the task manager and the python kernel must be quit. If a Results folder for this specific run has already been created, it must be deleted.

# Kmer Tiling

## Making enrichment dictionaries

After the raw data have been processed into kmers with associated enrichment, epitopes can be determined. The first step in doing this is loading kmer data into a dictionary, and storing that dictionary as a pickle. Be careful with module imports when pickling objects; if an object is created and then a module is re-imported, it won’t be able to be pickled.

directory = r'C:\KTOPE\Results\Example_Kmers’

kmerinfo = KmerInfo(directory)

kmerinfo.initialize_kmerinfo()

kmerinfo.make_enrichdicts()

This process will make a new pickled dictionary for each kmer file which is saved with the suffix “Enrichment_Dictionary.pickle”. This file will necessarily be larger than the kmer file from which it was made. By default, all kmer files are made into dictionaries e.g. 4def_4len.txt, 4def_5len.txt, etc. To make sure only specific kmer files are loaded, specify an additional kmerfiles argument in initializing the KmerInfo object:

directory = r'C:\KTOPE\Results\Example_Kmers’

kmerfiles = [‘4def_4len.txt’,’5def_5len.txt’]

kmerinfo = KmerInfo(directory,kmerfile=kmerfiles)

kmerinfo.initialize_kmerinfo()

kmerinfo.make_enrichdicts()

If it is desired to pickle the KmerInfo object itself, it won’t contain the enrichment dictionaries, to keep its size small.

## Generating RandomStats

To attach statistical significance to epitopes, random proteins need to be examined to get baseline statistics. Generally 10,000 random proteins are used which means that can take a long time to generate the statistics.

To start, a list of “all proteins” needs to be downloaded. This list is acquired by going to uniprot.org and searching all reviewed proteins or:
<http://www.uniprot.org/uniprot/?query=*&fil=reviewed%3Ayes>

Go to “Download” and make sure “Download all” and “Format: FASTA (canonical” are chosen. This may take a while since it’s a big file. Alternatively, a subset of this list can be downloaded.

Make this list into a pickled object using the commands (using previous kmerinfo object):

numproteins = 10000

allproteinfile = r'C:\KTOPE\Data\ uniprot-all-reviewed.fasta’

randomstats = RandomStats(kmerinfo,numproteins)

randomstats.make_all_proteins(allproteinfile)

These commands make a (large) pickled file with all proteins. This needs to be done only once.

Assuming this has been done already, a new RandomStats object can be initialized which loads in this pickled list, picks random proteins, and generates statistics.

numproteins = 10000

allproteinpickle = r'C:\KTOPE\Data\all_proteins.pickle’

randomstats = RandomStats(kmerinfo,numproteins,allproteinpickle=allproteinpickle)

randomstats.random_protein_search()

This will generate a file with ‘_RandomStats.pickle’ appended to the directory name. Make sure all parameters you use to make the RandomStats object match the parameters you use in finding epitopes later. The parameters available to set are:

epiminlen (int): min length for epitopes. Default: 6

epimaxlen (int): max length for epitopes. Default: 15

seqmin (int): minimum length of sequences used. Should be equal

to at least 2 times the number of maximum positions. If sequence

is too short, there is no way to tile it. Default: 10

randomseed (int): seed for random number generator. Default: 0

nameaddendum (str): suffix of the randomstats file in case multiple

randomstats files are generated for the same directory. Default: ‘’

spanval (int): window size to average the frequency for each

position. Must be odd. Default: 7

edgeweight (int): value that end positons in window are weighted.

Positions closer to center are linearly weighted with center

value being weighted as 1. Default 0.1

## Determining epitopes

First load the relevant KmerInfo object (uses load_enrichdicts rather than make_enrichdicts):

directory = r'C:\KTOPE\Results\Example_Kmers’

kmerinfo = KmerInfo(directory)

kmerinfo.initialize_kmerinfo()

kmerinfo.load_enrichdicts()

Next, load a protein of interest:

accession = ‘P0C0I3’

protdirectory = r'C:\KTOPE\Proteins"

protein = Protein(accession=accession,protdirectory=protdirectory)

protein.load_or_search()

This will search the protein online using BioPython and pickle the protein. If the protein has already been pickled, it will be loaded with these commands.

Alternatively, a protein can be loaded from a fasta file to avoid using the internet:

protein = Protein.parse_fasta(fastafile)

#Pickle protein after it has been read from fasta

protein.pickle_protein()

Fasta must have correct format downloaded from uniprot.org as FASTA (canonical). If its not in this format, protein name, organism and accession will not be correct.

To find epitopes, use the commands:

proteinepitopes = ProteinEpitopes(protein,kmerinfo)

proteinepitopes.initialize_proteinepitopes()

Optional parameters used in the ProteinEpitopes object creation are:

specificsamples ([Sample]): list of samples to be analyzed. If not

specified, all samples are used. These samples can be taken from

kmerinfo.samples so that they are in the correct format.

epiminlen (int): min length for epitopes. Default: 6

epimaxlen (int): max length for epitopes. Default: 15

randomstats (RandomStats): RandomStats pickle corresponding to data.

Will be loaded automatically if not specified but for examining

multiple proteins, it’s better to only load it once.

nameaddendum (str): suffix of the randomstats file in case multiple

randomstats files are generated for the same directory. Default: “”

Also initialize_proteinepitopes has an option calcstatistics which by default is set to True. If set to false, then percentiles won’t be calculated, which could be useful if you haven’t calculated RandomStats.

To view epitopes that have been found, first figure out the sample number corresponding to the sample of interest. This can be accomplished using KmerInfo’s sample2num function. Then access that key in the proteinepitopes epitopedict dictionary.

samplenum = kmerinfo.sample2num[Sample.parse_sampleline('Project Classification Name')]

proteinepitopes.epitopedict[samplenum]

This will display a list of epitopes for the sample:

[KAGQKTDDMLNS 116.71 (60, 72) 99.91% Streptolysin O (Streptococcus pyogenes serotype M1),

SNDVEAAFSAA 38.12 (357, 368) 74.19% Streptolysin O (Streptococcus pyogenes serotype M1),

NKVVTKDF 23.57 (401, 409) 71.33% Streptolysin O (Streptococcus pyogenes serotype M1)]

The epitopes are displayed with the epitope sequence, its score, the interval in the protein sequence in which it’s found (inclusive, exclusive), its percentile using RandomStats (if available), and the protein of interest. The list is ranked by percentile.

## Determining consensus epitopes

To find epitopes that are common to a population, the consensus_epitope command is used. Because this involves clustering, it takes significantly longer than finding each sample’s individual epitopes.

#percentile cutoff

epitopethresh = 95

#Minimum prevalence of the epitope in the population

minprev = 0.3

proteinepitopes.consensus_epitopes(epitopethresh,minprev)

Then the consensus epitopes can be called:

proteinepitopes.consensusepitopes

Giving:

[QKTDDMLNS 69.88 (63, 72) 0.421 Streptolysin O (Streptococcus pyogenes serotype M1)]

Here, there is also a number between the epitope’s location in the sequence and the protein’s name (0.421) which corresponds to the prevalence of the epitope in the population.

Other parameters that can be used in consensus_epitopes are:

randomseed (int): seed for RNG for kmeans. Default: 0

clusterdistance (int): minimum distance epitope centroids must

be from each other. Default 10

njobs (int): number of iterations in each K-means iteration. Default: 10

It may be desired to decrease clusterdistance to find epitopes that are closer together, though this may also make the list more redundant.

Note: The SciKit Kmeans algorithm used for find consensus epitopes is multi-threaded and automatically determines the number of processors to use.

proteinepitopes.epitopescores can be called to give the percentile and score for consensus epitopes, indexed by epitope objects.

## Determining epitopes for a list of proteins

Finding epitopes for a list of proteins is really just applying the algorithm multiple times. To facilitate the process, the ProteomicEpitopes class can be used. To start, a file with an accession on each line is used. This file can be compiled manually or obtained on uniprot.org by searching proteomes with desired filters. First, the object needs to be initialized and proteins need to be loaded in.

accessionfile = r'C:\KTOPE\Data\accession_list.txt"

protdirectory = r'C:\KTOPE\Proteins"

minprev = 0.3

epitopethresh = 95

randomstats = RandomStats.load_randomstats(kmerinfo)

proteomicepitopes = ProteomicEpitopes(kmerinfo,protdirectory, minprev,epitopethresh,randomstats,accessionfile=accessionfile)

proteomicepitopes.load_proteins_accessions()

This command will load all of the proteins corresponding to accessions from Biopython and could thus fail if there is a problem with your internet connection. Once all proteins used are downloaded from uniprot.org, they can be simply loaded as pickles.

Note: Since proteins are loaded from online, if there is an error with your internet connection the download may fail. However, since all proteins up to that point were downloaded, it should be able to quickly re-load them by running load_proteins again. Downloading many proteins may take a long time.

Alternatively, the proteins can be loaded from a single FASTA file downloaded from uniprot.org in FASTA (canonical) format:

fastafile = r'C:\KTOPE\Data\fasta_list.txt"

protdirectory = r'C:\KTOPE\Proteins"

minprev = 0.3

epitopethresh = 95

randomstats = RandomStats.load_randomstats(kmerinfo)

proteomicepitopes = ProteomicEpitopes(kmerinfo,protdirectory, minprev,epitopethresh,randomstats,fastafile=fastafile)

proteomicepitopes.make_proteins_fasta()

This will load in each protein from the fasta file and pickle it. This only needs to be run once for a set of proteins, since after that an accession file can be used to load from pickled proteins.

RandomStats can also be loaded using the nameaddendum argument in addition to kmerinfo if the RandomStats pickle has a suffix. The additional parameters available for initializing proteomicepitopes are the same as for RandomStats and ProteinEpitopes: epiminlen, epimaxlen, specificsamples, seqmin, spanval, and edgeweight.

To determine epitopes, there are two options, proteomic_individual_epitopes and proteomic_consensus_epitopes. The individual option compiles all epitopes that meet the epitopethresh parameter for each sample. The consensus option finds all consensus epitopes meeting minprev and epitopethresh and compiles them into a list. Naturally, the consensus option will take much longer to run.The progress in finding epitopes is displayed in the console as ‘Searched X% of proteins’ where ‘X’ is a multiple of 10.

They are run as follows:

proteomicepitopes.proteomic_individual_epitopes()

And the epitopes can be seen using the individualepitopes dictionary which functions the same as the epitopedict dictionary for ProteinEpitopes.

proteomicepitopes.proteomic_consensus_epitopes()

And the epitopes can be seen using the consensusepitopes attribute.

proteomicepitopes.epitopescores can be called to give the percentile and score for consensus epitopes, indexed by epitope objects.

To remove redundancy in the epitope list:

similarityfile = r'C:\KTOPE\Data\PAM30.txt"

similarity = Similarity(similarityfile)

cutoff = 10

nonredepitopes = similarity.non_redundant(proteomicepitopes.consensusepitopes, cutoff)

## Identifying group-specific epitopes

First load kmer data that is relevant for all samples you want to analyze.

directory = r'C:\KTOPE\Results\Example_Kmers’

kmerinfo = KmerInfo(directory)

kmerinfo.initialize_kmerinfo()

kmerinfo.load_enrichdicts()

randomstats = RandomStats.load_randomstats(kmerinfo)

Next, define the samples that will be used in each group. Each sample must be initialized from the Sample class i.e. using Sample(name,classification,project).

expgroup = [SampleE1, SampleE2 ,…, SampleEN]

controlgroup = [SampleC1, SampleC2, …, SampleCN]

Specify the protein directory, epitope threshold, prevalence, and specificity parameters. Then initialize the GroupCompare object.

protdirectory = r'C:\KTOPE\Proteins"

expthresh = 95

controlthresh = 80

minprev = 0.3

minspec = 1.0

groupcompare = GroupCompare(kmerinfo,protdirectory,expthresh,controlthresh,minprev,minspec,randomstats,expgroup,controlgroup)

For identifying group-specific epitopes a single protein, load the protein and then use the single_protein_compare function.

accession = 'P06484'

protein = Protein(accession=accession,protdirectory=protdirectory)

protein.load_or_search()

groupcompare.single_protein_compare(protein)

And then you can access the group-specific epitopes using the groupcompare.specificepitopes attribute.

For identifying group-specific epitopes for entire proteomes, specify an accession or fasta file.

accessionfile = r'C:\KTOPE\Data\accession_list.txt"

fastafile = r'C:\KTOPE\Data\fasta_list.txt"

Then identify epitopes using the multiple_protein_compare command to identify group-specific epitopes.

groupcompare.multiple_protein_compare(accessionfile=accessionfile)

OR

groupcompare.multiple_protein_compare(fastafile=fastafile)

Finally, access the newly identified epitopes using groupcompare.specificepitopes.
